# Mapping the insomnia patient journey in Europe and Canada

**DOI:** 10.3389/fpubh.2023.1233201

**Published:** 2023-08-29

**Authors:** David O’Regan, Diego Garcia-Borreguero, Fenna Gloggner, Imane Wild, Chrysoula Leontiou, Luigi Ferini-Strambi

**Affiliations:** ^1^Sleep Disorders Centre, Guy’s Hospital, London, United Kingdom; ^2^Faculty of Life Sciences and Medicine, King’s College, London, United Kingdom; ^3^Sleep Research Institute, Madrid, Spain; ^4^Idorsia Pharmaceuticals Ltd, Allschwil, Switzerland; ^5^Sleep Disorders Center, Università Vita-Salute San Raffaele, Milan, Italy

**Keywords:** insomnia, patient experience, patient journey, qualitative interviews, quality of life, quantitative survey

## Abstract

**Introduction:**

Insomnia affects daily functioning and overall health, and is thus associated with significant individual, societal, and economic burden. The experience of patients living with insomnia, their perception of the condition, and its impact on their quality of life is not well documented. The objective of this study was to map the patient journey in insomnia and identify unmet needs.

**Methods:**

Participants were individuals with insomnia, and healthcare professionals (HCPs) who treat insomnia, in the United Kingdom, France, Germany, Italy, and Canada. Qualitative interviews (50 patients, 70 HCPs) and a quantitative survey (700 patients, 723 HCPs) were conducted to inform the patient-journey mapping and obtain information on the emotions, perceptions, and experiences of patients and HCPs.

**Results:**

The patient journey comprises seven phases. The first defines the onset of insomnia symptoms. Phase 2 represents self-initiated behavior change to improve sleep (e.g., sleep hygiene, reducing caffeine, exercise). The next phase is characterized by use of over-the-counter (OTC) treatments, which generally fail to provide lasting relief. Phase 4 describes the first HCP consultation (occurring several months to several years after onset) and typically occurs at a crisis point for the patient; patients may be looking for an immediate solution (e.g., medication), which may not align with their HCP’s recommendation. The following stage comprises sleep hygiene/behavioral changes (±OTC treatment) under HCP guidance for many patients, although offering prescription treatments without a sleep hygiene stage under supervision is more common in some countries. Phase 6 describes prescription medication initiation, where patients fluctuate between relief/hopefulness and a sense of failure, while HCPs try to balance the need to provide relief for the patient while maintaining best medical practice and minimizing adverse effects. The final phase (living with long-term insomnia) represents an indefinite period during which sleep issues remain unresolved for many patients, with most of them continuing to use prescription treatments for longer than indicated and creating their own variable, self-managed regimens combining multiple modalities.

**Conclusion:**

This patient journey analysis for insomnia revealed seven distinct phases, highlighting different touchpoints where insomnia management could be optimized.

## 1. Introduction

Sleep health is essential for good physical and mental health (1, 2), and an unsatisfactory quantity and/or quality of sleep directly impacts patients and their quality of life. Some of the well-known consequences of sleep difficulties or disorders, such as insomnia, are impaired daytime functioning and cognition (3, 4), decreased workplace productivity (5, 6), and injuries and accidents (7–9). Acute insomnia is often triggered by acute stressors and is generally transient, resolving when the stressor ends (10). By contrast, chronic insomnia has a duration of at least 3 months, according to the Diagnostic and Statistical Manual of Mental Disorders, Fifth Edition (DSM-5; termed insomnia disorder) (11) and the International Classification of Sleep Disorders-Third Edition (ICSD-3; termed chronic insomnia disorder) (12), and it is defined as dissatisfaction with sleep quantity or quality (difficulty initiating or maintaining sleep, or early-morning awakening) occurring at least three times per week that causes clinically significant distress or functional impairment. Chronic insomnia may be triggered by chronic stress exposure and perpetuated by maladaptive coping strategies (10). The worldwide prevalence of chronic insomnia is high, affecting 6–10% of the adult population, with women more often affected than men and a greater frequency observed in older adults (10, 13, 14). Chronic insomnia is associated with serious health conditions such as psychiatric disorders, cardiovascular disease, diabetes, and Alzheimer’s disease (10, 15–19), with sleep being added as an essential pillar of cardiovascular health by the American Heart Association in 2022 (20). Given its effects on daily functioning and overall health, chronic insomnia is associated with significant individual, societal, and economic burdens (21–26).

Despite its high prevalence, insomnia is often under-recognized and insufficiently treated by non-expert healthcare professionals (HCPs) (27–31), who may have limited educational resources and understanding of the overall impact of this condition, especially when it becomes chronic (31, 32). Chronic insomnia is often not recognized by HCPs as a standalone condition; generally, the focus is on treating comorbid conditions and expecting the insomnia to resolve rather than treating insomnia directly (27, 31, 33, 34). This may stem from the historical classification of “primary” and “secondary” insomnia, which are no longer used in the DSM-5 and the ICSD-3; chronic insomnia is now classified as a standalone disorder independently of the presence or absence of comorbidities. It may also reflect a lack of HCP education on sleep disorders (33, 35, 36). Chronic insomnia also poses a dilemma for clinicians and health authorities because the disorder is defined by a duration of at least 3 months, but the use of most common pharmacological treatments is limited to 4 weeks, including down-titration (10, 37). Consequently, there are no effective and well-tolerated pharmacological treatments approved for chronic insomnia. Melatonin is approved for up to 13 weeks in patients ≥55 years of age, but its efficacy is deemed low by guidelines (10, 37, 38). Cognitive behavioral therapy for insomnia (CBT-I) can be used over the long term and shows sustained efficacy; however, up to 40% of patients do not achieve remission (39), and access and adherence to CBT-I can also be challenging (40–42). Patient acceptance of CBT-I as a management option for chronic insomnia may also be low due to time constraints and long waiting times to receive treatment (40). Thus, HCPs are faced with a difficult choice in the treatment of chronic insomnia, particularly when patients do not respond to the first-line treatment of CBT-I. In such cases, HCPs can either propose no further treatment, leaving patients without a solution for their chronic insomnia, or prescribe gamma-aminobutyric acid receptor agonists (GABA-RAs) off-label (i.e., beyond 4 weeks) (43–45), thus increasing the risk of adverse effects such as dependency (46). Other complex approaches involving experimenting with off-label treatments such as antidepressants (23), or switching between treatments every 4 weeks (47), may also occur.

Little attention has been paid to patients’ experience of living with insomnia, their perception of the condition, and its impact on their quality of life. Thus, as for many other disorders, the patient perspective on chronic insomnia and its treatment is not well documented (32). This may explain the difference in perceptions and expectations between patients and HCPs for this disorder (29, 32). Patients report thinking of insomnia as a consequence of their lifestyle (e.g., high levels of life stress, lack of sleep hygiene) rather than as a medical disorder and consequently feel responsible for their inability to achieve satisfying sleep. Interactions with primary care providers (PCPs) may reinforce such feelings, as patients are commonly advised to simply practice sleep hygiene in order to improve their sleep (32, 34, 48). Patients also report stigma related to their chronic insomnia, which makes it difficult for them to share their experiences of the disorder with others (48). Patient journey mapping is a commonly applied method for assessing the overall patient experience. Patient journeys can capture insights into patient perceptions, feelings, and motivations throughout their healthcare journey as a series of sequential steps. Patient journeys can also reveal areas where unmet needs exist, identify key stakeholders, and highlight how the management of a condition can be improved (49–51). Here we describe an exploratory study that mapped the patient journey in insomnia using data from qualitative interviews and a quantitative survey with both individuals with insomnia and HCPs who treat insomnia in Europe and Canada. A social media listening study provided complementary insights into the insomnia patient journey.

## 2. Methods

The work was conducted as part of a broader market research analysis to obtain insights relevant to insomnia. The qualitative interview guide and the quantitative survey questionnaire were designed and developed by an independent market research agency in collaboration with Idorsia Pharmaceuticals Ltd. The objective was to obtain information on the emotions, perceptions, and experiences of patients with insomnia and of HCPs who treat patients with this disorder. Pilot interviews and surveys were conducted to confirm that the instruments captured the type of data being sought. Participants were recruited by the market research agency and screened for eligibility prior to recruitment using a screening questionnaire. Participants were ineligible if they or a family member were employed by a pharmaceutical company/market research company or companies active in related fields. Participants were also excluded if they had taken part in market research on the topic of insomnia in the preceding 3 months. There was no overlap of participants between the qualitative and quantitative studies. Participants were informed of their rights and asked to provide consent at the screening stage as well as before the beginning of the interview/survey. In qualitative interviews, respondents were informed of the name of the study sponsor (Idorsia) at the end of their interview, in line with European Union General Data Protection Regulation requirements. A separate, unrelated social media listening study on insomnia was also undertaken.

### 2.1. Qualitative interviews

Qualitative interviews were conducted in Europe (United Kingdom [UK], France, Germany, Italy) and Canada from January to February 2021 by the market research agency and their local subcontractors in the local language. Interviews were conducted by telephone with online screen sharing with patients and HCPs. Patient interviews were 75 min and HCP interviews were 60 min in length. Patient eligibility criteria were as follows: age 30–70 years; diagnosed with insomnia with an Insomnia Severity Index (ISI) (52) score of ≥8; must rate at least one sleep-related symptom as moderate or above [score of 2 or greater on a severity scale ranging from 0 (none) to 4 (very severe)]; had a consultation with an HCP for insomnia within the past 3 months for initial interviews (later relaxed to 6 months to facilitate recruitment); and must be currently taking a drug treatment [prescription or over-the-counter (OTC)]. The research agency ensured that time since first insomnia consultation varied among the selected participants. Patients with a diagnosis of obstructive sleep apnea, chronic pain, schizophrenia, post-traumatic stress disorder, or bipolar disorder were excluded. The following HCP screening criteria were used: PCP, sleep specialist, neurologist, or psychiatrist with 3–30 years in practice; ≥75% of professional time spent in direct patient care; ≥30 adult patients with insomnia seen in a typical month; and ≥ 15 patients on prescription therapy (must prescribe a mixture of drug classes). Additional criteria were applied to ensure an appropriate distribution of HCPs according to practice type (i.e., hospital/office), practice setting (community and university/teaching hospital-based), and private/public practice. Both patient and HCP interviews addressed the topics of experience of living with insomnia, attitudes and perceptions toward insomnia, treatment of insomnia, and treatment patterns and goals.

### 2.2. Quantitative surveys

Quantitative surveys were conducted with both patients and HCPs; different questionnaires were used for each population. Surveys were conducted in Europe (UK, Germany, France, Italy, Spain) and Canada via a 30-min online questionnaire in the local language from April to May 2021. A range of different question types was utilized, including questions with precoded answers (single and multiple), open questions, and statement pairs to be answered using a 1–7-point sliding scale.

For the patient quantitative survey, participants were screened for eligibility at the beginning of the survey using the following criteria: age 30–70 years; selected insomnia from list of health conditions without prompting; had subthreshold, moderate severity, or severe insomnia [ISI score ≥ 8 points when administered during screening; maximum of 40 patients with subthreshold insomnia (score 8–14) to be included]; taking prescription or OTC medication (required quota of 60 and 40%, respectively), with those receiving OTC being naïve to prescription treatments (quota 20%) or prescription treatment-lapsed (quota 20%); and had a consultation for insomnia with an HCP within the past 6 months. Patients with obstructive sleep apnea, chronic pain, bipolar disorder, or schizophrenia were excluded. Topics for the patient survey included living with insomnia (knowledge/impact/self-management), patient–doctor relationship, and treatments and medications (general perceptions/current treatment).

For the HCP survey, inclusion criteria were as follows: PCP, sleep specialist, neurologist, or psychiatrist having been in current specialty/role for 3–35 years; spend at least 70% of time seeing patients; see at least 30 adult patients with insomnia in a typical month and treat at least 15 with prescription therapy per month. Additional criteria were applied to ensure the appropriate representation according to practice type (hospital/office), practice setting (community, university/teaching), and proportion of private/public patients; quotas varied by country and by specialty. Topics for the HCP survey were attitudes toward and relationship with patients, number of insomnia patients treated, time spent treating them, and the characteristics of these patients, perceptions of insomnia, and attitudes toward insomnia management and treatment.

The quantitative survey questionnaires are included in Supplementary Appendix.

### 2.3. Social media listening

The passive social media listening study was based on monitoring social media (including Twitter, blogs, forums, YouTube, and photo-sharing sites) for spontaneous opinions and discussions in relation to insomnia. The aim was to understand patient experiences with treatment and factors that influence treatment decisions, and pain points and concerns were identified. The social media listening was conducted from January to December 2019 by a third-party vendor on behalf of Idorsia using social listening tools. Keywords, phrases, and hashtags associated with insomnia along with their translations in local languages were used to aggregate the data. Analysis of the collected data was conducted after removal of news articles and irrelevant content to identify trends and concerns. Listening was conducted in the UK, Spain, Italy, Germany, and France, and languages were English, Spanish, Italian, German, and French. All data were obtained from publicly accessible sources and were anonymized.

### 2.4. Patient journey mapping

The information obtained in the quantitative and qualitative studies was integrated in the analysis to identify key touchpoints between the patient and the care system during their quest for solutions to their insomnia, and to develop a map of the patient journey in insomnia. Information from the social media listening study was also reviewed to supplement the journey mapping.

## 3. Results

### 3.1. Key findings from patient interviews and surveys

The characteristics of the 50 patients (10 from each of the five countries) included in the qualitative interviews are shown in Table 1. In interviews, patients reported that insomnia had a significant negative impact on their sense of self and felt it had consequences for their concentration, day-to-day functioning, and mental wellbeing. Insomnia was regarded by patients as a personal inability to do something that they considered natural and that should be easy to achieve. Patients also had the general belief that insomnia was “something they should be able to fix,” which fueled anxiety and caused feelings of guilt. Patients reported that initially, they tried to self-manage their insomnia (e.g., lifestyle changes, sleep hygiene measures, OTC treatments) and cycled through the various options. Seeking external help was seen as a failure for some. At the time of first medical consultation for insomnia, patients reported that they were seeking a solution for their disorder that would help them sleep rather than more advice around management strategies such as mindfulness methods, sleep hygiene measures, and OTC treatments that they may have already tried. At this stage they may be ready to accept a medical solution. Interview findings suggested that patients generally had low expectations from treatments, anticipating and accepting suboptimal outcomes. Medication dependency was a key concern, and feedback suggested that patients felt responsibility to reduce or stop insomnia treatment. Few patients reported being recommended or prescribed CBT-I.

**Table 1:** Characteristics of patients included in qualitative interviews.

Characteristic	UK (*n* = 10)	France (*n* = 10)	Germany (*n* = 10)	Italy (*n* = 10)	Canada (*n* = 10)	Total number of patients (*N* = 50)
Age, years^1^						
Mean	NA^2^	49.5	56.1	47.9	NA	
Range	30–70^2^	34–66	33–68	33–65	30–70^2^	
Gender, n						
Female	5	7	7	6	5	30
Male	5	3	3	4	5	20
ISI total score category, n						
Subthreshold (8−14)	2	2	2	0	1	7
Moderate severity (15−21)	4	6	6	5	5	26
Severe (22−28)	4	2	2	5	4	17
Use of medication, n						
Prescription medication	7	8	9	9	9	42
OTC medication	3	2	1	1	1	8

^1^A minimum of 50% of patients included were to be ≥50 years of age.

^2^Age was reported by selecting the appropriate age bracket; actual ages were not recorded.

ISI, Insomnia Severity Index; NA, not available; OTC, over-the-counter; UK, United Kingdom.

**Figure 1:**
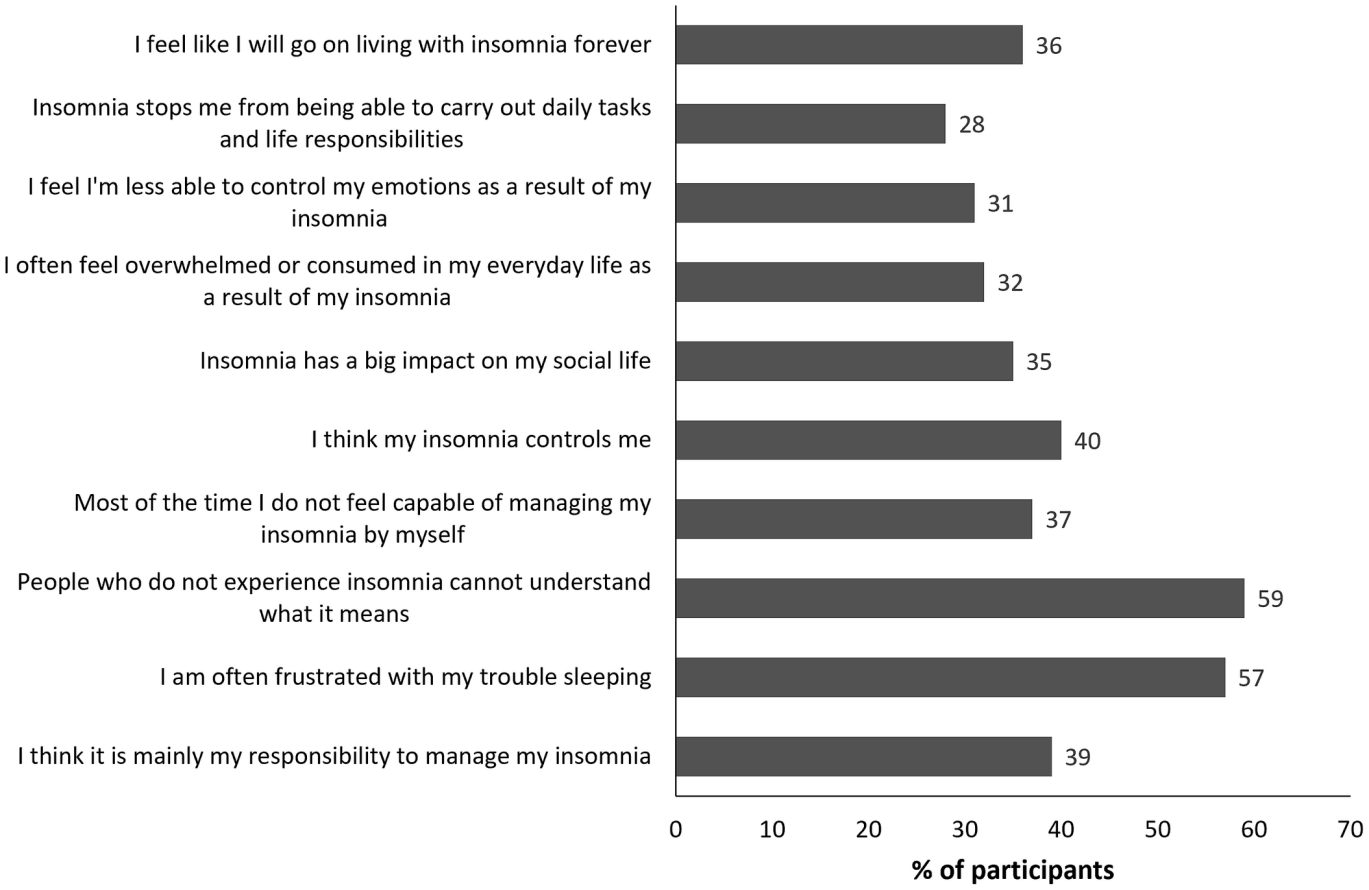
Quantitative survey questions on the impact of insomnia on patients’ daily life that are relevant to the patient journey. Values shown reflect the percentage of respondents selecting the highest 2 scores (6 or 7) on a 1- to 7-point scale where 7 indicates the strongest agreement.

**Figure 2:**
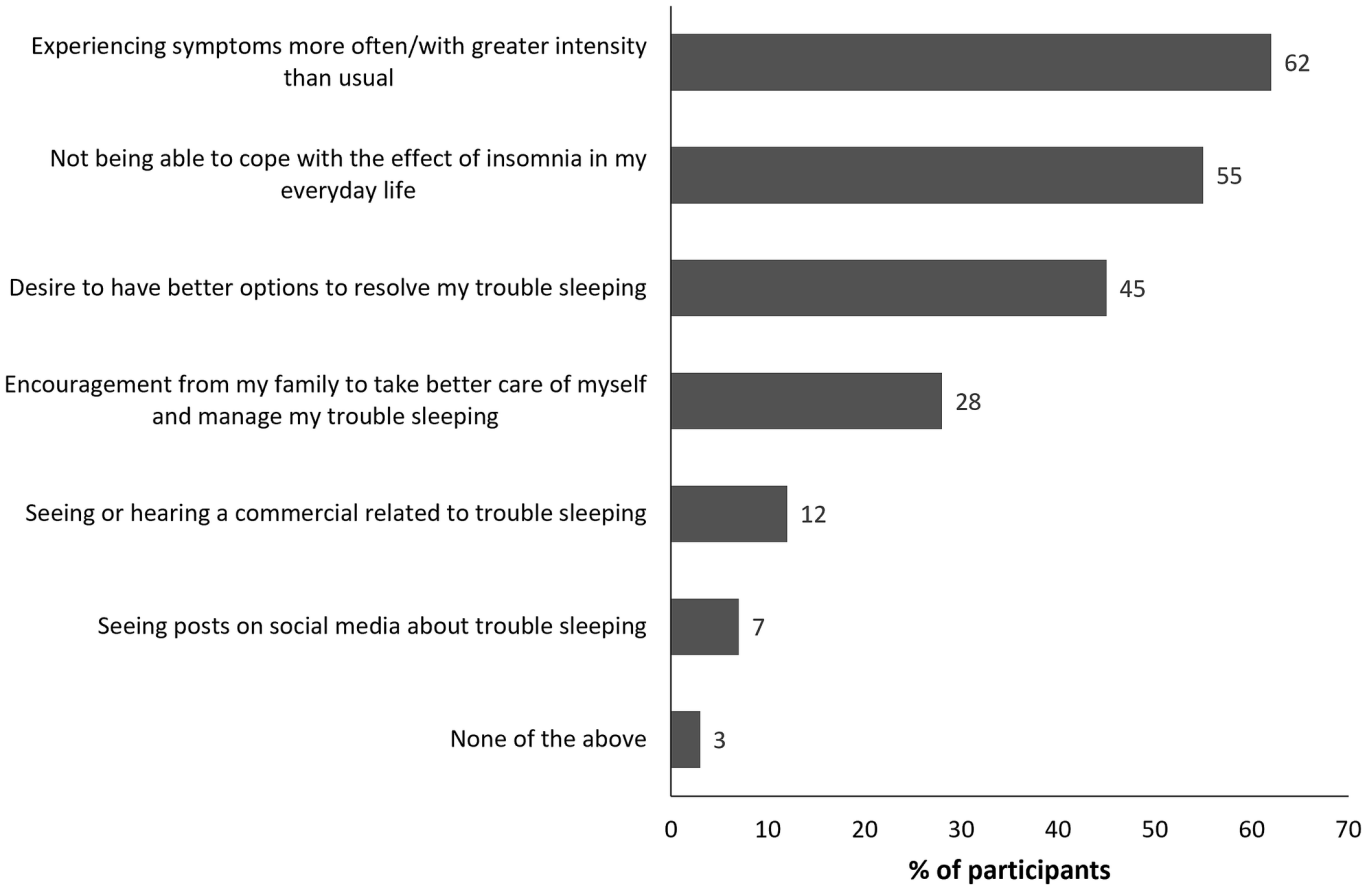
Quantitative survey question on drivers for seeking help with insomnia. Values shown reflect the percentage of patients selecting each answer.

Characteristics of the 700 patients who completed the quantitative survey are shown in Table 2. The mean age was 46.7 (standard deviation 10.5) years and 60% were female. The first consultation with an HCP to discuss trouble sleeping occurred at least 3 months prior to the survey in 92% patients, and at least 4 months prior in 89%. The mean length of time since the first consultation for insomnia was 5.9 years. Moderate to severe insomnia was present in 93% of patients (ISI score 15–28). Selected data from the surveys based on relevance to the patient journey are provided in the main manuscript text, tables, and figures; additional data are available in the Supplementary Appendix. Patients reported that insomnia had a significant negative impact on their personal wellbeing and social life; selected survey answers on this topic are shown in Figure 1. Overall, 57% of patients surveyed reported that they were often frustrated with their sleep problems and 59% believed that those who do not experience insomnia could not understand its impact. Overall, 28% of patients indicated that insomnia impacted their ability to carry out daily life tasks and responsibilities. Among the surveyed patients, 32% reported often feeling overwhelmed or consumed in everyday life because of insomnia, and 31% considered themselves less able to control their emotions. Additionally, 40% of patients felt controlled by their insomnia and 37% felt that most of the time they were not capable of managing it by themselves. Despite the latter point, 39% thought it was mainly their own responsibility to manage their insomnia. Only 31% of patients felt that their insomnia was an independent medical problem not influenced by other health issues. Key drivers for seeking help from HCPs were an increase in the frequency and intensity of insomnia symptoms (62% of patients) and inability to cope with symptoms such as fatigue and memory issues and their impact on everyday life (55% of patients) (Figure 2). Having a positive relationship with their HCP was important to 71% of patients, but only 30% of patients felt that their HCPs were the main decision-makers regarding treatment of their insomnia. The mean (standard deviation) number of consultations before patients received their first prescription medication was 2.6 (2.7); however, this varied by country, being 4.1 in Germany and 2.0 in Italy. Overall, 27% of patients received their first prescription medication at their first consultation and 41% received it within 2–5 consultations; this differed substantially from the overall population in Germany (8% at first consultation and 36% within 2–5 consultations) and the UK (13% at first consultation, 49% within 2–5 consultations). However, the number of patients who did not know how many consultations they had before receiving prescription medication was higher in these countries (Germany 47% and UK 35% versus 28% overall). Of the surveyed patients, 34% reported wanting to stop medication but were fearful of not being able to sleep. Only 7% of patients reported using CBT-I in the past 3 months. Discussions with their PCPs were the most frequent source of information on insomnia in 90% of patients.

**Table 2:** Characteristics of patients included in quantitative survey.

Characteristic	All patients (*N* = 700)
Gender, n (%)	
Female	419 (60)
Male	281 (40)
Age, years	
Mean (SD)	46.7 (10.5)
Median	46.0
Minimum, maximum	30, 70
Number of comorbidities	
Mean (SD)	2.3 (1.1)
Comorbidities occurring in > 20% of patients, n (%)	
Anxiety	304 (43)
Migraines	183 (26)
Mild–moderate depression	191 (27)
ISI score	
Mean (SD)	20.9 (3.9)
ISI category, n (%)	
Subthreshold (8–14)	47 (7)
Moderate severity (15–21)	325 (46)
Severe (22–28)	328 (47)
Use of prescription medication, n (%)	
Naïve	93 (13)
Current user	412 (59)
Lapsed user	195 (28)
Time since first consultation for insomnia, years	
Mean	5.9
Time since first consultation for insomnia category, n (%)	
1–2 months	57 (8)
≥3 months	643 (92)
≥4 months	621 (89)
Time since last consultation in days	
Mean (SD)	54.5 (50.2)
Employment status, n (%)	
Working full- or part-time	549 (78)
Not working	90 (13)
Retired	59 (8)
Prefer not to say	2 (<1)
Level of education, n (%)	
No formal qualifications	4 (1)
Trade apprenticeship	92 (13)
High school or equivalent	177 (25)
Higher education below degree level	102 (15)
Undergraduate degree	186 (27)
Postgraduate degree	138 (20)
Country, n (%)	
Canada	115 (16)
France	115 (16)
Germany	110 (16)
Italy	120 (17)
Spain	120 (17)
UK	120 (17)

ISI, Insomnia Severity Index; OTC, over-the-counter; SD, standard deviation; UK, United Kingdom.

### 3.2. Key findings from HCP surveys and interviews

**Figure 3:**
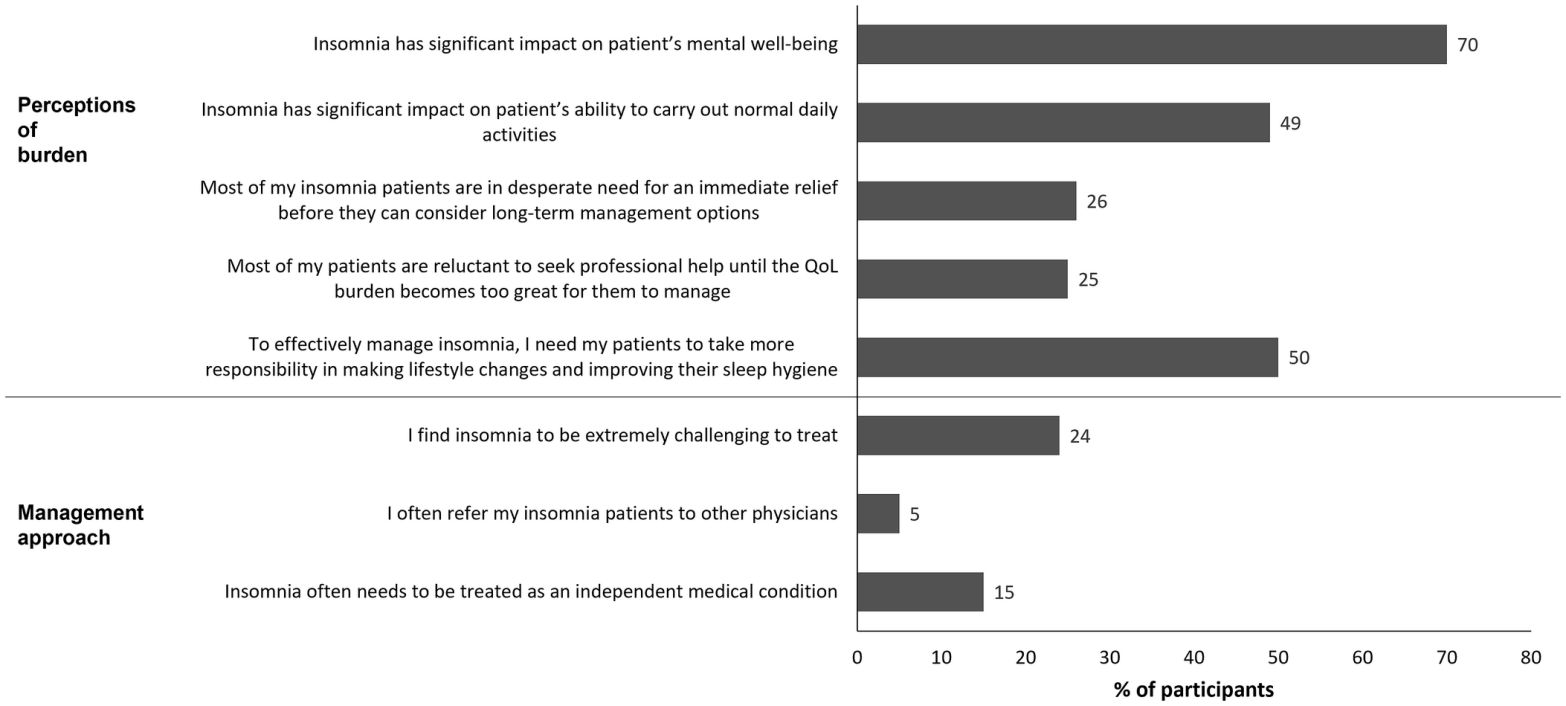
Quantitative survey questions on HCP perceptions of insomnia and management approach that are relevant to the patient journey. Values shown reflect the percentage of respondents selecting the highest 2 scores (6 or 7) on a 1- to 7-point scale where 7 indicates the strongest agreement. HCP, healthcare professional; QoL, quality of life.

Qualitative interviews included 70 HCPs (40 PCPs and 30 sleep specialists/neurologist/psychiatrists)—8 PCPs and 6 specialists from each country. The quantitative HCP survey included 723 individuals (PCPs 60%, sleep specialists 6%, neurologists 7%, and psychiatrists 27%); their characteristics are shown in Table 3. Selected survey questions around HCP perceptions of insomnia and management approach based on relevance to the patient journey are shown in Figure 3. There was some discordance between the HCP and patient survey outcomes, particularly in relation to responses on insomnia and its impact on patients. Although some patients felt their HCP was empathetic to their insomnia experience, others believed that their treating physician did not understand the full impact of the condition on them. Among surveyed HCPs, less than half (49%) had insight into the significant impact that insomnia had on their patients’ ability to complete normal daily activities. There was also a misunderstanding of the stage at which patients seek help, with the majority of HCPs not realizing that patients were usually at a crisis point when they contacted them. This contrasts with the findings of the qualitative interviews with patients, which suggested that most patients were at crisis point by the time of their first medical consultation for insomnia. Only 26% of HCPs appreciated the urgency for immediate relief from insomnia before considering long-term management options. Interview findings suggested that although CBT-I was seen as an ideal option by around half of HCPs, it was perceived as unrealistic; reasons for this included difficulty in access, level of commitment required, and patient reluctance for fear of mental health connotations. In the survey, 23% of HCPs reported offering CBT-I at first consultation, which was less frequent than offering prescription medication (37%) and suggesting OTC treatment (26%). Only 5% of HCPs stated that they often refer their patients with insomnia to other physicians. At first consultation for insomnia, HCPs only suggested referral to a psychologist in 17% of patients and referral to another specialist in 13% of patients. Nonetheless, 24% of HCPs surveyed acknowledged that they found insomnia extremely challenging to treat; this was particularly true in the UK (39%), Germany (29%), and Canada (28%).

**Table 3:** Characteristics of HCPs included in quantitative survey.

Characteristic	All HCPs (*N* = 723)
Gender, n (%)	
Female	248 (34)
Male	449 (62)
Age, years	
Mean (SD)	47.8 (10.1)
Median	48.0
Minimum, maximum	27, 69
Primary specialty, n (%)	
Neurologist	48 (7)
PCP	434 (60)
Psychiatrist	195 (27)
Sleep specialist	46 (6)
Years of experience	
Mean (SD)	17.2 (8.6)
Percentage of time in clinical practice	
Mean (SD)	91.0 (7.6)
Personal experience with insomnia, n (%)^1^	
Yes	400 (55)
No	323 (45)
Personal experience with insomnia treatment, n (%)^1^	
Yes	299 (75)
No	73 (18)
Prefer not to answer	28 (7)
Primary setting, n (%)^2^	
Hospital	139 (23)
Office	464 (77)
Number of patients with insomnia under care	
Mean (SD)	600.1 (318.1)
Number of adult patients with insomnia in 1 month	
Mean (SD)	88.6 (80.1)
Proportion of patients by category, %	
Chronic insomnia (>3 months)	61
Acute insomnia (<3 months)	29
Unknown duration	10
Proportion of patients with insomnia being treated for mental health condition	
%	56
Number of patients with insomnia personally prescribed therapy	
Mean (SD)	72.1 (67.7)
Percentage of time treating insomnia	
Mean (SD)	24.2 (20.3)
Country, n (%)	
Canada	121 (17)
France	121 (17)
Germany	121 (17)
Italy	120 (17)
Spain	120 (17)
UK	120 (17)

^1^Self or close relative/friend.

^2^Excludes UK-based physicians (denominator = 603).

HCP, healthcare professional; PCP, primary care provider; SD, standard deviation; UK, United Kingdom.

There were some areas of concordance between HCPs and patients regarding unmet needs. Most HCPs (70%) recognized that insomnia had a significant impact on patients’ mental wellbeing. HCPs expressed dissatisfaction with current treatments, with only 13% being satisfied with existing treatment options for chronic insomnia. HCPs also expressed concern about the risks associated with available treatments (e.g., adverse effects such as dependence), and the lack of long-term treatments with a good safety profile. The lack of long-term options for some patients was stated by 64% of HCPs as one of the main challenges with regard to prescription medication. When queried about the most important attributes of prescription medication for chronic insomnia, the top three categories selected by HCPs were “does not cause dependency” (54%), “safety for long-term use” (44%), and “ability to improve daytime functioning” (42%). As with patients, HCPs did not often perceive insomnia as a primary disorder that was independent of other medical conditions, with only 15% strongly agreeing that it often needs to be treated as an independent medical condition.

### 3.3. Social media listening findings

The observational social media listening findings confirmed the findings from the patient surveys and interviews. The findings showed that insomnia significantly affected the daily functioning of patients (education, work, and social life), and patients highlighted the limitations of existing treatments. The major common concerns raised in relation to all the currently available drug classes were fear of addiction, inefficacy, and adverse effects. There was some dissatisfaction expressed in the social media listening results regarding the HCP–patient relationship.

### 3.4. Describing the patient journey in insomnia

**Figure 4:**
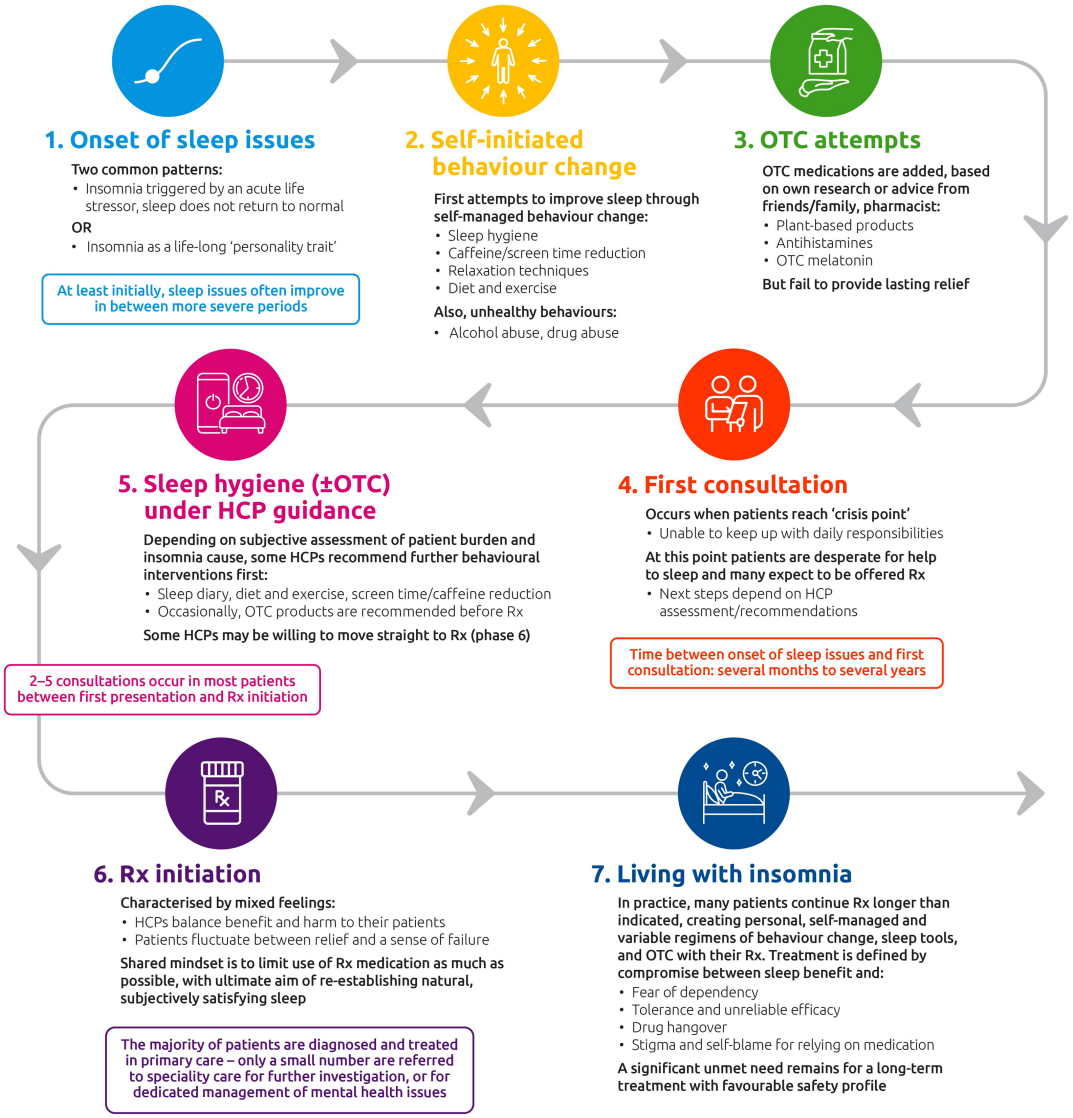
The insomnia disorder patient journey. HCP, healthcare professional; OTC, over-the-counter; Rx, prescription treatment.

The seven phases of the patient journey in insomnia that were identified are shown in Figure 4. The typical journey is expected to differ slightly by geography based on differences in healthcare systems (53). Furthermore, the journey may not be linear, and some patients may oscillate between different phases for a long time before progressing, or may even regress.

Phase 1 describes the onset of sleep issues. In the qualitative research, two patterns were commonly described: (1) the insomnia was first triggered by an acute life stressor, but after the stressor passed, sleep did not return to normal; or (2) the insomnia was described as something patients had experienced for most of their lives and was perceived to be part of their personality: “It’s just who I am.” At least initially, patients report that their sleep issues often improve in between more severe periods. Even in this initial phase, patients described their insomnia as having a negative impact on sense of self, and how they are able to relate to others. Patients commonly describe their insomnia in terms of a personal failing and an inability to achieve sleep, rather than as a medical condition. Furthermore, some patients and HCPs perceive insomnia to be predominantly a symptom of other underlying conditions or the result of lifestyle choices and it thus lacks the status of a full medical condition.

In Phase 2 (self-initiated behavior change), patients describe making initial attempts to improve sleep through self-managed changes in behavior (e.g., sleep hygiene, reducing caffeine and screen time, relaxation, diet, and exercise). Unhealthy behaviors such as use of alcohol or drugs are also occasionally mentioned. At this stage, most patients try to deal with insomnia on their own without seeking medical help in an effort to avoid admitting to themselves that their insomnia is a serious problem. Seeking help is sometimes associated with a sense of failure in solving their sleep problem by themselves.

Phase 3 (OTC attempts) is often characterized by starting the use of OTC sleep treatments chosen by the patient based on their own research or based on advice from friends/family or a pharmacist. OTC options may include plant-based products, antihistamines (in countries where a prescription is not needed), and non-prescription melatonin if available. However, these products generally fail to provide lasting relief and patients may cycle through multiple OTC treatments as they try to find a solution. At this stage, patients commonly describe complex “sleep regimens” consisting of specific behaviors and a variety of different sleep aids, including OTC treatments and additional items such as special pillows, candles, or sleep apps.

The time from onset of sleep issues to Phase 4 (first consultation) can range from several months to several years. The first HCP consultation for insomnia typically occurs at a crisis point: patients describe a sense of desperation as their established sleep routines and interventions fail to provide relief. The problem of “not sleeping” becomes one of “not coping” as patients feel increasingly unable to keep up with their day-to-day responsibilities. At this stage, many patients are looking for an immediate solution that will actually help them sleep after having tried many strategies already (e.g., behavior changes, sleep hygiene) and are ready to accept a medical solution. At this point, they often expect to at least be offered medication, which may not align with their HCP’s recommended course of action. Most patients have their first consultation in primary care and remain in this setting for treatment – only a small number are referred to specialty care for further investigation or for dedicated management of mental health issues although access to a specialist varied by country.

For many patients, Phase 5 comprises sleep hygiene (±OTC treatments) treatments under HCP guidance. Depending on subjective assessment of patient burden and insomnia cause, some HCPs recommend further behavioral interventions before moving to prescription medications. Sleep hygiene measures and other behavioral changes (e.g., in diet and exercise) are usually the recommended measures, and OTC products are occasionally recommended. Some HCPs may be willing to try prescription treatments immediately (Phase 6), without this Phase 5 step as an initial measure. However, most patients have 2–5 consultations before they receive prescription medication; this varies by country.

Phase 6 (prescription medication initiation) is characterized by mixed feelings for both the patient and their HCP. Patients fluctuate between relief/hopefulness and a sense of failure in taking the “easy way out” with prescription treatment, while HCPs describe trying to balance the need to provide relief for the patient while maintaining best medical practice and minimizing adverse effects of prescription treatment. Patients are often aware of a strong association between some of the currently available GABA-RAs and dependency, and this is a key barrier to acceptance. Likewise, HCPs aim to limit the use of GABA-RA medication as much as possible while re-establishing natural, subjectively satisfying sleep.

The final stage (Phase 7—living with long-term insomnia) represents an indefinite period during which sleep issues remain unresolved for most patients. In practice, many patients continue to use prescription treatments for longer than indicated, creating their own self-managed and variable regimens combining behavior change, sleep tools, and OTC medication with their prescription treatment. Longer-term use of prescription drugs is defined by a compromise between sleep benefit and fear of dependency, as well as issues around tolerance and unreliable efficacy, drug hangover, and its impact on daily functioning. Several patients also reported feeling stigma/self-blame for having to rely on medication to achieve sleep.

## 4. Discussion

The results of the extensive qualitative interviews, quantitative surveys, and social media listening steps of this work illustrate the perceptions and attitudes of patients and HCPs toward insomnia and its treatment, and they provide insights into the concordance/discordance between patient and HCP perceptions and expectations regarding its management. The knowledge obtained from these initiatives was subsequently synthesized to create a conceptual model of the patient journey in insomnia, which can be described as defined by unmet needs and compromises. This patient journey analysis revealed seven distinct phases, highlighting different touchpoints that may enable HCPs to better understand patients’ needs at each stage and determine an optimal management approach for each patient. In the quantitative survey patient population, the mean length of time since the first consultation was 5.9 years and the first consultation with an HCP to discuss insomnia occurred at least 3 months prior to the survey in 92% of patients, suggesting that the majority had long-term insomnia.

Pharmacological treatment plays a key role in insomnia management but is currently suboptimal. Currently available drugs (e.g., GABA-RAs) have a warning to limit their use to 4 weeks maximum to minimize side effects and risk of dependency (10, 37), which does not align with the unmet need for longer-term treatment in patients with chronic insomnia. Insomnia thus places HCPs in a frustrating situation in which they are trying to find a balance between providing relief for the patient and maintaining best medical practice (e.g., not prescribing off-label treatment and exposing their patient to the risk of drug dependency) (34, 54). The qualitative interviews in this study revealed that both HCPs and patients recognized dependency as a risk of prescription medications. Both prompted (interviews and survey) and unprompted feedback (social media listening) from patients also showed significant concern about dependency. However, HCPs may resort to off-label long-term prescription of these medications, with patients accepting this as a necessary risk to be relieved from their insomnia (33, 43, 44, 55). Thus, negative effects during the day, such as impaired daytime functioning (46, 56), could become normalized. Furthermore, the common desire to limit the use of sleep medications as much as possible could result in patients self-managing complex regimens that comprise multiple modalities (e.g., sleep hygiene plus OTC plus prescription medication) (57), the combination of which might change nightly based on their day-to-day activities; this may cause anxiety and thus perpetuate insomnia.

Several stages of the patient journey, in particular diagnosis and treatment success evaluation, are characterized by subjectivity both in terms of patient reports and of HCPs’ treatment choice. This is ultimately what matters in chronic insomnia because clinical evaluation relies on subjective criteria in daily clinical practice (10, 11), particularly at the PCP level. Additionally, the patient journey may differ subtly according to geographic region given the differences in healthcare systems. The journey of patients with insomnia was also made more challenging by the COVID-19 pandemic because access to HCPs was reduced (58).

Here we report the comprehensive patient journey in insomnia from the patient viewpoint. Our findings affirm and significantly build on those of smaller qualitative and focus group studies evaluating experiences and perceptions of insomnia management in primary care (21, 29, 33, 34, 36, 59). The strength of our study lies in the substantially larger patient and HCP populations included compared with these previous studies, which have included <30 patient or HCP participants. Our work combines research findings from three studies and includes both qualitative and quantitative components, whereas others have included only qualitative assessments. Furthermore, our study includes populations from multiple countries, whereas others report data from a single country (21, 29, 33, 34, 36, 59). The findings of these smaller studies broadly align with each other and with our findings. Selection bias is a potential limitation in our study and may have resulted in lack of representation, because participants were recruited on criteria chosen according to internal considerations of the sponsor, and no attempt was made to reflect specific insomnia populations by country. Furthermore, data were not stratified by insomnia severity or duration, and no subgroup analyses were conducted to explore possible differences according to participant characteristics such as sex/gender or age. Social listening relies on the directness of patients and HCPs and the assumption that the information provided is authentic; furthermore, only patients with a certain level of health literacy and computer fluency participate in such forums, which again creates a selection bias.

The finding in the current study that patients may be initially reluctant to consult an HCP has been described by others (29, 57, 60, 61). Our finding that insomnia lacked the status of a full medical condition for patients and HCPs also aligns with previous findings (27, 29, 33, 34, 54), although one survey of Spanish PCPs found that 89% considered insomnia to be an important health problem (62). This provides an opportunity to educate both groups on the impact of the disorder and its status as a primary medical condition, and the importance of seeking help earlier to avoid the crisis point at which many patients find themselves when initiating contact with their HCP. Indeed, the International Classification of Diseases 11th Revision newly recognizes chronic insomnia as a disorder (63). Insomnia may not be perceived as a serious disorder, possibly because it does not usually appear to affect mortality (64). However, it is associated with an increase in unintentional fatal injuries (7), and in the long term, insomnia is associated with serious health conditions such as psychiatric disorders, cardiovascular disease, diabetes, and neurodegenerative diseases (10, 16–19), which may be life-shortening. Disorders that do not impact mortality [e.g., migraine (65)] but significantly impair the life of patients are accepted as medical conditions that need treatment by both patients and PCPs. Insomnia has similar implications for the patient and should be treated on the same level. The lack of acceptance of insomnia as a primary disorder by HCPs may diminish the relevance it is given and impact the treatment plan, e.g., result in a short treatment course that does not solve the problem, and perpetuates suboptimal management.

Insomnia may also be perceived by HCPs as difficult to diagnose, but it can be readily diagnosed with the correct criteria (10, 56). A contributing factor may be the limited time that PCPs in public health services have for each patient consultation, being insufficient to properly diagnose and manage insomnia (34). HCPs may also feel that there is a lack of education and training for them in sleep medicine (33, 36, 54, 66), which ties in with the lack of referral to specialists (34, 36, 62) and potentially to patients giving up trying to access help. Unprompted feedback (social media listening) showed sometimes less-than-optimal patient–HCP interactions, which may reflect HCP training gaps and the perceived difficulties in diagnosing insomnia, although it should be noted that social media findings may be biased (67) (e.g., people tend to share either very good or very bad experiences online) (68). These findings show scope for improvement in HCP education and training around sleep disorders.

Overall, our findings show that patient education is needed to remove the stigma around insomnia, to help patients recognize that chronic insomnia is a primary disorder requiring appropriate medical management, and to encourage them to seek help sooner. HCP education and training on sleep disorders are also needed to increase the perception of chronic insomnia as a serious primary medical condition, and to fully understand the impact this disorder has on the daily life of their patients. Furthermore, the current situation in which patients have to be satisfied with “manageable” insomnia highlights a serious unmet need regarding management and treatment. This has previously been highlighted in a survey of patients with insomnia in the UK, which found that 76% still had insomnia symptoms after 12 months despite receiving primary care management or prescription treatment (69). The patient journey described here has not yet been validated; this will be an area for future research. Additional research with a representative sample from each country and the use of more stringent methodology are needed to strengthen these findings.

## 5. Conclusion

The patient journey in insomnia is slow and characterized by suboptimal choices for both patients and HCPs, highlighting several unmet needs. Insomnia significantly impacts patients’ wellbeing, social life, and daily functioning both prior to pharmacological treatment and after receiving treatment. At the initial stages of their journey, patients with insomnia are reluctant to consult an HCP and often suffer for long periods before seeking help. Patients self-manage complex treatment regimens and behaviors comprising multiple modalities and need to make decisions on sleep management every night, which increases their anxiety. When they finally consult an HCP, their condition has progressed to a crisis point. CBT-I is the recommend first-line therapy in treatment guidelines but access to it, and acceptance/adherence is limited. Pharmacological options are suboptimal and are not indicated for long-term treatment. At the maintenance stage of the patient journey, an effective solution for managing symptoms is typically not reached. Patients and HCPs often settle for suboptimal outcomes and do not demand more from their treatment choices.

## Data availability statement

The original contributions presented in the study are included in the article/Supplementary material, further inquiries can be directed to the corresponding author.

## Ethics statement

Ethical review and approval was not required for the study on human participants in accordance with the local legislation and institutional requirements. Written informed consent for participation was not required for this study in accordance with the national legislation and the institutional requirements.

## Author contributions

DO’R, DG-B, and LF-S contributed to interpretation of the data, revision of the manuscript critically for important intellectual content, and approval of the final version. FG contributed to the conception and design of the work, analysis of the data, interpretation of the data, revision of the manuscript critically for important intellectual content, and approval of the final version. IW and CL contributed to the conception and design of the work, revision of the manuscript critically for important intellectual content, and approval of the final version. All authors contributed to the article and approved the submitted version.

## Conflict of interest

This work was supported by Idorsia Pharmaceuticals Ltd., Allschwil, Switzerland. This study sponsor was involved in designing the interview questionnaires and surveys (in collaboration with third-party market research vendors); in the analysis and interpretation of data; in the writing of the report; and in the decision to submit the article for publication. Data collection was conducted by the third-party vendors. DO’R, DG-B, and L-FS were independent of the funder. All authors had full access to all the data in the study and can take responsibility for the integrity of the data and the accuracy of the data analysis. DO’R has received honoraria for lectures, presentations and/or participation in scientific advisory boards from the British Association of Psychopharmacology, Neurodiem, TEVA, and Idorsia. DG-B has received research grants from MSD and Roche, and has performed consulting work for Idorsia and Roche. FG, IW, and CL are employees of Idorsia Pharmaceuticals Ltd. LF-S has received honoraria for lectures, presentations and/or participation in scientific advisory boards from Sanofi, Lundbeck, Italfarmaco, Valeas, Angelini, Bayer, Pfizer, Bioprojet, Jazz Pharma, and Idorsia.

## Publisher’s note

All claims expressed in this article are solely those of the authors and do not necessarily represent those of their affiliated organizations, or those of the publisher, the editors and the reviewers. Any product that may be evaluated in this article, or claim that may be made by its manufacturer, is not guaranteed or endorsed by the publisher.
