# Supplementary material for: Mapping the insomnia patient journey in Europe and Canada
Source: Front Public Health. 2023 Aug 29;11:1233201. doi: 10.3389/fpubh.2023.1233201 (PMC10497771; doi:10.3389/fpubh.2023.1233201)
Supplement: Supplementary file 1 [file Data_Sheet_1.docx]

**Supplementary Appendix**

Mapping the insomnia patient journey in Europe and Canada

David O’Regan, Diego Garcia-Borreguero, Fenna Gloggner, Imane Wild, Chrysoula Leontiou, Luigi Ferini-Strambi

Contents

[Patient quantitative survey questionnaire 1](#_Toc123821979)

[HCP quantitative survey questionnaire 19](#_Toc123821980)

[Results from patient quantitative survey 28](#_Toc123821981)

[Results from HCP quantitative survey 37](#_Toc123821982)

# Patient quantitative survey questionnaire

Note: The survey also included a series of questions related to an investigational product, described as “Product X”, which are not shown here.

**SECTION A: General attitudes**

**A4.** Please read pairs of emotions below and tell us where you would place yourself

between adjectives to describe your perception of your overall mental health.

(Please focus on how you feel in general, irrespective of the current COVID-19 situation)

[PN: DO NOT SCRIPT “Mental health”]

1. Concerned | | At peace
2. Vulnerable | | Protected
3. Worried | | Confident
4. Alone | | Supported
5. Overwhelmed | | In Control
6. Confused | | Reassured
7. Guilty | | Blameless
8. Pessimistic | | Optimistic

[PN: SLIDING SCALE, PLEASE START AT NEUTRAL. RANDOMIZE PAIRS]

**A5.** To what extent do you agree with each of the following statements about your effort in conducting a healthy life?

(Please select one answer only per each statement)

[PN: TOP, RESPONSES]

1. Strongly disagree
2. Neither agree nor disagree
3. Strongly agree

**[PN: LEFT, STATEMENTS]**

1. Of all the things I can do to maintain good health, eating well is the most important
2. Of all the things I can do to maintain good health, sleeping well is the most important
3. I exercise regularly to keep in good physical shape
4. I go to the regular health checks that my doctor recommends
5. I take vitamin and mineral supplements, or traditional herbs and spices

**[PN: SINGLE CODE PER STATEMENT. RANDOMIZE STATEMENTS]**

**A6.** To what extent do you agree with each of the following statements about your health?

(Please select one answer only per each statement)

[PN: TOP, RESPONSES]

1. Strongly disagree
2. Neither agree nor disagree
3. Strongly agree

**[PN: LEFT, STATEMENTS]**

**[PN: DO NOT SCRIPT “General attitude towards health”]**

1. I do as much as I can to promote and maintain my personal health and wellness
2. I prefer alternative/holistic approaches rather than standard medical practices
3. I do not seek help from doctors unless I am very sick
4. I try to stay well informed about my health
5. I am better informed about my health than most people
6. I often talk honestly about my mental health with my friends and family
7. I prefer brand name medications to generic ones
8. I would rather bear moderate to severe pain than treat my condition with a prescription medication
9. I am willing to make any lifestyle changes necessary to avoid having to take a prescription medication
10. I am not willing to tolerate side effects from my prescription medication(s)
11. I prefer to treat myself with an over-the-counter medication than to depend on a doctor to give me a prescription medication
12. Avoiding products that are bad for my health is more important than buying products that are good for my health
13. I will sacrifice convenience if it means getting better medications
14. I am willing to try new treatments even if they have yet to be proven as effective over a long period

[PN: SINGLE CODE PER STATEMENT. RANDOMIZE STATEMENTS]

A7. How is your health in general, would you say?

1. Very poor
2. Very good

[PN: SINGLE CODE]

**SECTION B: Living with Insomnia**

INTRO. Now we would like to talk to you about your experience of living with insomnia.

B1. Which of the following expressions, would you use to define what “good sleep” means to you?

(Please select up to 5 expressions that you would use)

1. Falling asleep within a short period of time from going to bed
2. Having undisturbed sleep
3. Waking up at a reasonable time, not very early in the morning
4. Sleeping without nightmares
5. Having more than a certain number of hours of sleep
6. Waking up feeling refreshed
7. Waking up feeling ready for the day
8. Waking up and feeling energetic all the next day
9. Waking up and not feeling drowsy all the next day
10. Waking up and looking rested and rejuvenated
11. Able to fall asleep naturally

[PN: RANDOMIZE. MULTICODE. SELECT UP TO 5 CODES. DO NOT FORCE]

**B2.** Which, if any, of the following ways do you currently use to monitor or have previously used to monitor your sleep quantity and quality?

(Please select all that apply for each period)

**[PN: TOP]**

- a. Use **currently**
- b. Used **in the past**

**[PF: LEFT]**

1. Smart Devices, such as FitBit, Smart Watches and Oura Ring
2. Sleep Tracking Apps downloaded onto my phone
3. A Diary (paper or digital)
4. Other (specify _______________)
5. None of the above [PN: EXCLUSIVE. **FIX]**

**[PN: RANDOMIZE. MULTICODE BOTH B2a AND B2b]**

B3. Please consider your insomnia and your experiences with it over time. Which of the following would you use to describe how often you experience insomnia?

For the purpose of this question, I would like you to consider your experience with measures or treatments already taken.

(Please select one answer)

1. **The insomnia I experience is constant**. This could be most nights of the week over a long period of time.
2. **The insomnia I experience comes and goes without always having a specific reason**. This could be for days or weeks at a time before it improves and then eventually returns.
3. **The insomnia I experience occurs at specific times.** This could be triggered during stressful events, or difficult times.
4. **I’m not sure how my insomnia changes over time**
5. **I don’t see a pattern with regards to how my insomnia comes and goes**
6. **My insomnia is bad for a short period of time, but then often goes away** for a long time as well
7. None of the above [PN: EXCLUSIVE. **FIX**]

**[PN: RANDOMIZE. SINGLE CODE]**

B4. Which of the following do you consider to be the **main reason for your current insomnia**? Please consider any potential triggers for your current insomnia.

(Please select all relevant answers)

1. Hectic lifestyle
2. Fear of nightmares
3. It’s just who I am
4. Racing thoughts at night
5. Recent stressful life events or circumstances, including relationships, work and COVID-19
6. Stressful experiences in my past
7. Other mental health issues
8. Poor sleep routine
9. Hard to unwind at night
10. Too much screen time on digital devices
11. A recent traumatic event, such as a bereavement
12. There is no apparent cause **[PN: AUTO SELECT IF CODE 2 SELECTED AT B3]**
13. I don’t know **[PN: EXCLUSIVE. FIX]**

**[PN: RANDOMIZE. MULTICODE. NOT TO SHOW IF CODE 2 SELECTED AT B3]**

B5**.** For the next questions please consider the impact of your insomnia.

Which part of your **next day** do you think is **most impacted** after a bad night’s sleep as a result of your insomnia?

(Please select only one answer)

1. Early morning
2. Late morning
3. Early afternoon
4. Late afternoon
5. Evening
6. Night/when you go to bed again

**[PN: SINGLE CODE]**

B6**.** Please read the following pairs of statements and indicate where you would place yourself on the scale below.

[PN: TOP, RESPONSES]

- Strongly disagree
- Neither agree nor disagree
- Strongly agree

[PN: LEFT, PAIRS OF STATEMENTS]

1. I find my insomnia stops me from being able to carry out daily tasks and life responsibilities **||** Despite my insomnia, I’m still able to continue my normal daily tasks and life responsibilities
2. I often feel overwhelmed or consumed in my everyday life as a result of my insomnia **||** Despite my insomnia, I still feel a reasonable degree of control in my everyday life
3. Insomnia has a big impact on my social life **||** Insomnia has little to no impact on my social life
4. I think my insomnia has caused me to become more reserved or introverted **||** I don’t think insomnia has any impact on how I am as a person
5. I feel I’m less able to control my emotions as a result of my insomnia **||** Despite my insomnia, I have a good control of my emotions
6. I feel like I will go on living with my insomnia forever **||** I feel like I will get through this period of insomnia and be able to sleep normally again soon

[PN: SLIDING SCALE, PLEASE START AT NEUTRAL, ONE PAIR AT A TIME. RANDOMIZE PAIRS]

B7. Please read pairs of statements below and indicate where you would place yourself

between statements*.*

1. I believe if I treat my other conditions, my trouble sleeping will resolve automatically | | I believe my trouble sleeping is an independent medical problem, not influenced by any other health issues
2. I believe my trouble sleeping should be treated in its own right, independently of any other health issues | | I believe all my health conditions should be considered and treated together, including my trouble sleeping

[PN: SLIDING SCALE, PLEASE START AT NEUTRAL]

B9. To what extent do you agree with the below statements?

(Please select one answer per statement)

[PN: TOP, RESPONSES]

- Strongly disagree
- Neither agree nor disagree
- Strongly agree

[PN: LEFT, STATEMENTS]

1. I am reluctant/self-conscious to admit to others that I have insomnia
2. I worry my insomnia is seen by others as a mental health issue
3. I am often frustrated with my trouble sleeping
4. If you are reading this, please select 7 [Data quality control]
5. I feel guilty about how my trouble sleeping affects people around me
6. I feel my insomnia is under control
7. People who do not experience insomnia cannot understand what it means
8. I feel supported by my family and friends when I need to manage my insomnia
9. Trouble sleeping is my biggest health concern
10. I am hopeful that my trouble sleeping will get better
11. I will probably always need prescription medications to manage my trouble sleeping

[PN: SINGLE CODE PER STATEMENT. RANDOMIZE STATEMENTS]

**SECTION C: Self-management of insomnia**

INTRO**.** For this section, we would like you to consider how you manage your insomnia.

C1. Thinking about when you first started having sleeping problems (before talking to a healthcare professional), what caused you to start thinking of your trouble sleeping as a problem?

(Please select the top 3 reasons)

1. Experiencing symptoms more often / with greater intensity than usual
2. Not being able to cope with the effect of insomnia in my everyday life
3. Encouragement from my family to take better care of myself and manage my trouble sleeping
4. Desire to have better options to resolve my trouble sleeping
5. Seeing or hearing a commercial related to trouble sleeping
6. Seeing posts on social media about trouble sleeping
7. None of the above [PN: EXCLUSIVE. FIX]

[PN: MULTICODE UP TO 3 CODES]

C2. How would you rate your **current** knowledge of insomnia (e.g. as a condition and its treatments)?

(Please select the option that best applies)

1. I have (almost) no knowledge about insomnia
2. I have a little knowledge about insomnia
3. I have some knowledge about insomnia
4. I have a lot of knowledge about insomnia

[PN: SINGLE CODE]

C3. When you were looking for information about insomnia, which of the following sources did you use or visit?

(Please select all that apply)

1. Discussion with my Primary Care Practitioner / General Practitioner
2. Discussion with a Specialist (e.g. Psychiatrist, Neurologist)
3. Online forums and blog posts
4. Social media (e.g. Facebook, Twitter, Instagram)
5. Specialist sleep websites
6. Discussion with family and friends
7. Online patient networks or support groups
8. Brochures found in my doctor’s office
9. Magazine / Newspaper articles
10. Books
11. Discussion with pharmacists
12. Discussion with alternative health practitioner
13. Discussion with staff at health shops (e.g. health food store, herbalist shop)
14. Other (please, specify _____) [PN: FIX]

[PN: RANDOMIZE. MULTICODE]

C4. And which sources of information do you find the most useful?

(Please select up to 3 sources)

[PN: LEFT, PIPE IN CODES SELECTED AT C3]

[PN: MULTICODE UP TO 3]

C5**.** Thinking about how you manage your insomnia, please indicate where you would place yourself

between the 2 opposite statements.

[PN: TOP, RESPONSES]

- Strongly disagree
- Neither agree nor disagree
- Strongly agree

[PN: LEFT, PAIRS OF STATEMENTS]

1. I feel completely in control of my insomnia **||** I think my insomnia controls me
2. I think it is mainly my responsibility to manage my insomnia **||** I think it is mainly the responsibility of my doctor to manage my insomnia
3. Most of the time I feel capable of managing my insomnia by myself **||** Most of the time I do not feel capable of managing my insomnia by myself
4. I am happy to take any prescription medication offered to me to get a good night’s sleep **||** Prescription medication would be the last resort for me

[PN: SLIDING SCALE, PLEASE START AT NEUTRAL, ONE PAIR AT A TIME – RANDOMIZE PAIRS]

C6. Which, if any, of the following are you currently using to manage your insomnia by yourself?

(Please select all that apply)

**C7**. Which, if any, of the following have you ever used to manage your insomnia by yourself?

(Please select all that apply)

**[PN: TOP, RESPONSES]**

- Using **currently [C6]**
- Used **previously [C7]**

[PN: LEFT, STATEMENTS]

[PN: DO NOT SCRIPT “Mindfulness activities”]

1. Cognitive Behavioural Therapy for Insomnia (CBT-I)
2. Yoga
3. Meditation
4. Breathing exercises
5. Other (specify ____________) [PN: FIX AT THE BOTTOM OF THIS SET]

[PN: DO NOT SCRIPT “Other measures”]

1. Alcohol
2. Cannabis (in any form)
3. Reducing caffeine intake
4. Reading before bed
5. Warm bath before bed
6. Not looking at phones or screens before bed
7. Running or other physical activities
8. Acupuncture
9. Weighted blanket
10. Other (specify __________) [PN: FIX AT THE BOTTOM OF THIS SET]
11. None of these [PN: FIX AT THE BOTTOM]

[PN: SHOW ONE SET AT A TIME AND ASK C6 AND C7, BEFORE MOVING TO NEXT ONE]

[PN: SHOW BEFORE C6 AND AFTER C7 – SAME ORDER AT C7]

[PN: RANDOMIZE SETS AND CODES WITHIN EACH SET]

[PN: MULTICODE BOTH C6 AND C7]

[PN: CREATE A VARIABLE TO IDENTIFY THOSE WHO USE MORE THAN ONE ALTERNATIVE REMEDY – MORE THAN 1 CODE SELECTED AT C6 BETWEEN 1 TO 14]

C8. Thinking about the measures that you are currently using to manage your insomnia, how frequently do you use them?

(Please selected one answer for each measure currently using)

I use it …

[PN: TOP, RESPONSES]

1. Every day
2. 5 to 6 times a week
3. 3 to 4 times a week
4. 1 to 2 times a week
5. Once a week
6. Less often than once a week
7. Once a month
8. Less frequently than once a month
9. Only when I need it/no specific frequency

[PN: LEFT, SHOW CODES SELECTED AT C6 ALTOGETHER]

[PN: SHOW C8 ONLY IF MORE THAN ONE BETWEEN 1 TO 14]

[PN: RANDOMIZE. SINGLE CODE PER ROW]

C10. Thinking about your **very** **first consultation** with a doctor to discuss your trouble sleeping, how long ago did it happen?

(Please enter the number of years OR months passed from your very first consultation)

- ______ years [PN: NUMERIC. RANGE 1–99]

OR

- ______ months [PN: NUMERIC. RANGE 1–99]

**C11.** Which of the following **motivated the decision** to see a doctor?

(Please select all that apply)

1. My insomnia got worse and I needed help
2. A friend or family member suggested I speak to my doctor
3. I had been suffering for a while and I needed help
4. My research of information sources made me think reaching out to a doctor would help
5. I had tried a lot of measures myself with little success
6. I wanted to see what the doctor would say
7. I wanted to know if something was wrong with me
8. I did not see the doctor for my insomnia, I booked a consultation for another reason and then we ended up discussing my sleep
9. Not being able to cope with the effect of insomnia in my everyday life
10. Encouragement from my family to take better care of myself and manage my trouble sleeping
11. Other (please, specify _____) **[PN: ANCHOR]**

[PN: RANDOMIZE. SINGLE CODE]

**SECTION D: Insomnia treatments**

**INTRO.** For this section, we would like you to consider your medications for insomnia.

[PN: ASK IF Rx LAPSED OR Rx USER]

D1. After how many times did you see your doctor about your trouble sleeping before your **first** **prescription medication** specifically for insomnia?

(By prescription medication we refer to medications that cannot be purchased without a prescription from a doctor)

(Please consider your first consultation to be 1)

- ______ [PN: NUMERIC. RANGE 1–25]
- I don’t know [PN: EXCLUSIVE CODE]

[PN: ASK IF Rx LAPSED OR Rx USER]

D2a. Which type of doctor prescribed your **first** **prescription medication** specifically for insomnia?

(Please selected only one answer)

1. Family doctor / Primary care physician / General practitioner
2. Neurologist
3. Psychiatrist
4. Sleep specialist
5. Psychologist
6. Internal medicine physician
7. Other (please specify ____________) [PN: FIX]

[PN: RANDOMIZE. SINGLE CODE]

[PN: ASK IF Rx LAPSED OR Rx USER]

D2b. Which type of doctor prescribed you any **subsequent repeat prescriptions of this medication** specifically for insomnia?

(Please select only one answer)

1. Family doctor / Primary care physician / General practitioner
2. Neurologist
3. Psychiatrist
4. Sleep specialist
5. Psychologist
6. Internal medicine physician
7. I have not had any subsequent repeat prescriptions [PN: ANCHOR]
8. Other (please specify ____________) [PN: FIX]

[PN: RANDOMIZE. SINGLE CODE]

[PN: ASK IF Rx LAPSED OR Rx USER]

D3. [ASK IF Rx PTS: If a **prescription medication** was offered to you, how willing would you be to take it?]

**[IF Rx LAPSED ASK:** When the **first** **prescription medication** was offered to you, how willing were you to take it?**]**

[PN: SHOW TO Rx LAPSED AND Rx PTS] By prescription medication we refer to medications that cannot be purchased without a prescription from a doctor.

[PN: SCALE]

- 1. Not at all willing
- 2.
- 3.
- 4.
- 5.
- 6.
- 7. Extremely willing

[PN: SINGLE CODE]

[PN: ASK IF Rx LAPSED OR Rx USER]

D4. When prescribed with your **first prescription medication**, what were your reasons for deciding to start taking it?

(Please select all that apply)

1. I felt that I had run out of other options
2. I needed something stronger to help me sleep
3. I couldn’t cope with my trouble sleeping
4. Advice from family and friends
5. I trusted the recommendation of the doctor I was seeing
6. I knew it would be only a short-term measure
7. I couldn’t cope with the impact insomnia was having on my life
8. Other, please specify ____________ [PN: FIX]
9. None of the above [PN: FIX. EXCLUSIVE]

[PN: RANDOMIZE. MULTICODE]

TO ALL

D5. **[PN: IF** OTC NAÏVE, SHOW: How would it make you feel if a prescribed medication was offered to you?**]**

**[PN: IF Rx LAPSED OR Rx USER, SHOW:** How did it make you feel when you were given your **first prescription medication**?**]**

(Please drag and drop the **5 emotions** that best apply to you)

**[PN: LEFT, ADJECTIVES]**

[PN: CONFUSED, DO NOT SCRIPT]

- 1. Conflicted
  2. Embarrassed
  3. Resigned
  4. Worried

[PN: SAD, DO NOT SCRIPT]

- 1. Guilty
  2. Disappointed
  3. Desperate
  4. Frustrated

[PN: STRONG, DO NOT SCRIPT]

- 1. Certain
  2. Secure
  3. Empowered
  4. Confident

[PN: HAPPY, DO NOT SCRIPT]

- 1. Happy
  2. Hopeful
  3. Optimistic
  4. Relieved

[PN: ANGER, DO NOT SCRIPT]

- 1. Annoyed
  2. Resentful
  3. Fed up
  4. Failure / Failed

[IF ONLY FOR OTC NAÏVE ADD CODE 99] The doctor has never offered me a prescription medication [EXCLUSIVE]

[PN: DRAG & DROP UP TO 5 CODES – RANDOMIZE AND SHOW ALL ADJECTIVES]

D6.

**[IF** OTC NAÏVE, SHOW]

How do you feel about taking a prescribed medication in the future?

**[IF Rx USER, SHOW]**

In general, how do you feel about the decision to take a prescribed medication to manage your insomnia **over a longer period**?

**[IF Rx LAPSED:]**

In general, how would you feel about starting a different prescription medication to manage your insomnia and taking it for a long period of time?

(Please select the statement that best applies to you)

**[PN: LEFT, STATEMENTS]**

[IF Rx USER, SHOW]

1. I have no hesitation to take prescription medication over a long period of time as long as it means that I can get a good sleep every night
2. I am ok to take prescription medication over a long period as long as I could stop when I want to and without having to deal with any withdrawal symptoms
3. I am ok to take prescription medication over a long period as long as I am able to manage any withdrawal symptoms and could stop when I want without becoming dependent
4. I have reservations about taking any prescription medication longer term, but I would take it if it was the only way I could get a good sleep every night
5. I only consider taking medication for a very short period and prefer to go back to managing my sleep with other measures as quickly as possible
6. I would **not** be willing to take prescription medication for a long period of time and I prefer to manage insomnia without prescribed medication at all
7. I’ve not considered this [PN: FIX]

[IF OTC NAÏVE OR Rx LAPSED, SHOW]

1. I would have no hesitation to take prescription medication over a long period of time as long as it meant that I can get a good sleep every night
2. I would be ok to take prescription medication over a long period as long as I could stop when I want to and without having to deal with any withdrawal symptoms
3. I would be ok to take prescription medication over a long period as long as I am able to manage any withdrawal symptoms and could stop when I want without becoming dependent
4. I would have reservations about taking any prescription medication longer term, but I would take it if it was the only way I could get a good sleep every night
5. I would only consider taking medication for a very short period and would prefer to go back to managing my sleep with other measures as quickly as possible
6. I would **not** be willing to take prescription medication for a long period of time and I would prefer to manage insomnia without prescribed medication at all
7. I’ve not considered this [PN: FIX]

[PN: RANDOMIZE. SINGLE CODE]

[PN: PLEASE USE THE SAME VARIABLE TO ALLOCATE THE ANSWERS PROVIDED]

D7. Thinking about your **[IF** OTC NAÏVE, SHOW **“current medication”, IF Rx USER “current prescription medication”, IF Rx LAPSED, SHOW** **“previous prescription medication”]**, to what extent do you agree with the following statements?

[PN: TOP, RESPONSES]

- Strongly disagree
- Neither agree nor disagree
- Strongly agree

[PN: LEFT, STATEMENTS]

[IF OTC NAÏVE OR Rx USER, SHOW]

1. My medication is the only thing that helps me to sleep
2. I use my medication as little as possible, only when it is really necessary
3. I avoid using my medication at all costs
4. I am keen to stop my medication soon, but I am worried that I could not sleep
5. I cannot imagine going on holiday/going away without my medication
6. If I have good sleep for a couple of nights, I will skip my medication
7. I always take medications following advice from my doctor / as prescribed
8. I am not comfortable talking about my medication with others around me

[IF Rx LAPSED, SHOW]

1. My medication was the only thing that helps me to sleep
2. I used my medication as little as possible, only when it was really necessary
3. I avoided using my medication at all costs
4. I was keen to stop my medication soon, but I was worried that I could not sleep
5. I could not imagine going on holiday/going away without my medication
6. If I had good sleep for a couple of nights, I skipped my medication
7. I always took medications following advice from my doctor / as prescribed
8. I was not comfortable talking about my medication with others around me

[PN: SINGLE CODE PER STATEMENT. RANDOMIZE STATEMENTS]

[PN: PLEASE USE THE SAME VARIABLE TO ALLOCATE THE ANSWERS PROVIDED]

D8. Thinking about your **[IF** OTC NAÏVE, SHOW **“current medication”, Rx USER “current prescription medication”, IF Rx LAPSED, SHOW “previous prescription medication”]**, **and your insomnia**, to what extent do you agree with the following statements?

[PN: TOP, RESPONSES]

- Strongly disagree
- Neither agree nor disagree
- Strongly agree

[PN: LEFT, STATEMENTS]

[IF OTC NAÏVE OR Rx USER, SHOW]

1. I feel that my insomnia can be effectively resolved in other ways
2. Even with my current insomnia medication, I cannot sleep properly
3. I feel my current insomnia medication is not the right one for me
4. If you are reading this, please select two [Data quality control]
5. I have a love-hate relationship with my insomnia medication
6. I would really like to take insomnia medications less often
7. I have asked my doctor about taking my insomnia medication less often

[IF Rx LAPSED, SHOW]

1. I felt that my insomnia could be effectively resolved in other ways
2. Even with my current insomnia medication, I could not sleep properly
3. I felt my current insomnia medication was not the right one for me
4. If you are reading this, please select two [Data quality control]
5. I had a love-hate relationship with my insomnia medication
6. I would really have liked to take insomnia medications less often
7. I had asked my doctor about taking my insomnia medication less often

[SINGLE CODE PER STATEMENT. RANDOMIZE STATEMENTS]

[PN: PLEASE USE THE SAME VARIABLE TO ALLOCATE THE ANSWERS PROVIDED]

**[PN: NEW SCREEN]** Now we would like talk about your **[IF** OTC NAÏVE, SHOW **“current medication”, Rx USER “current prescription medication”, IF Rx LAPSED, SHOW “previous prescription medication”]**, for trouble sleeping.

Previously you said that your current medication is: **[PN: PIPE IN “CURRENT TREATMENT” VARIABLE CREATED AFTER S5c]**

Previously you said that your most recent medication is **[PN: PIPE IN “PREVIOUS TREATMENT” FROM S5d]**

Please answer the next questions thinking about the medication shown above.

**[SCREEN END]**

D9. [SHOW TO NON-Rx LAPSED] How long have you been taking your **[IF** OTC NAÏVE, SHOW **“current medication”, Rx USER “current prescription medication”]**?

**[IF Rx LAPSED, SHOW**: How long were you taking your **previous prescription medication** for?

I have been taking **[PIPE IN “CURRENT TREATMENT” FOR OTC NAÏVE AND Rx USER]** for:

**[IF Rx LAPSED, SHOW**: I took **[PIPE IN PREVIOUS TREATMENT FROM S5d]** for:

- ______ days [NUMERIC. RANGE 0–30]

OR

- ______ months [NUMERIC. RANGE 0–12]

OR

- ______ years [NUMERIC. RANGE 0–99]

1. I don’t remember [PN: EXCLUSIVE]

D10. **[SHOW TO NON-Rx LAPSED]** How often do you **typically** take your **[IF** OTC NAÏVE, SHOW **“current medication”, IF Rx USER, SHOW “current prescription medication”]** for trouble sleeping?

**[IF Rx LAPSED, SHOW**: How often did you typically take your **previous prescription medication** for trouble sleeping?

(Please select one answer)

Your current medication is: **[PN: PIPE IN “CURRENT TREATMENT” VARIABLE CREATED AFTER S5c]**

**[IF Rx LAPSED, SHOW**: Your previous medication was: **[PIPE IN “PREVIOUS TREATMENT” FROM S5d]**

**[PN: LEFT, OPTIONS]**

1. Every night
2. 5 to 6 times a week
3. 3 to 4 times a week
4. 1 to 2 times a week
5. Once a week
6. Less often than once a week
7. Once a month
8. Less frequently than once a month
9. Only when I need it/no specific frequency
10. Other [PN: ANCHOR]

[PN: SINGLE CODE]

D11a. **[NON-Rx LAPSED]** Which of the following factors influence the frequency in which you take your **[IF** OTC NAÏVE, SHOW **“current medication”, IF Rx USER, SHOW “current prescription medication”]**?

**[IF Rx LAPSED USERS SHOW]** Which of the following factors influence the frequency in which you took your **previous prescription medication?**

(Please select all that apply)

Your current medication is: **[PN: PIPE IN “CURRENT TREATMENT” VARIABLE CREATED AFTER S5c]**

**[IF Rx LAPSED, SHOW**: Your previous medication was: **[PIPE IN “PREVIOUS TREATMENT” FROM S5d]**

1. Suggestion/recommendation by my doctor
2. I am worried about becoming dependent on medication
3. I am worried about other medications I am taking at the same time
4. My trouble sleeping does not occur every day
5. After a very stressful day
6. Before an important day
7. After having several nights of bad sleep
8. I take it freely and happily whenever I need it
9. Other, please specify ____________ [PN: ANCHOR]

**[PN: MULTICODE. RANDOMIZE]**

[PN: NOT TO ASK IF OTC NAÏVE]

D11b. [IF Rx USER, SHOW] Thinking about when you first received your **current prescription medication** for trouble sleeping, what advice or recommendations, if any, did your HCP give you regarding how frequently you should take it?

[IF Rx LAPSED, SHOW] Thinking about when you received your previous prescription medication for your trouble sleeping, what advice of recommendations, if any, did your HCP give you regarding how frequently you should take it?

(Please select the answer that’s most relevant to you)

- The doctor said I could take it daily within a set period of time (e.g. over a two-week period)
- The doctor said I should take it only when I need it
- The doctor said I should take as few as possible
- The doctor did not give any instructions regarding how to take them
- I don’t remember

[PN: SINGLE CODE]

D12. **[IF NON-Rx LAPSED]** Thinking specifically about your **[IF** OTC NAÏVE, SHOW **“current medication”, IF Rx USER, SHOW “current prescription medication”]**, how effective is it for helping you with your trouble sleeping?

**[IF Rx LAPSED, SHOW]** Thinking specifically about your **previous prescription medication**, how effective was it for helping you with your trouble sleeping?

(Please place yourself on the scale below)

Your current medication is: **[PN: PIPE IN “CURRENT TREATMENT” VARIABLE CREATED AFTER S5c]**

**[IF Rx LAPSED, SHOW**: Your previous medication was: **[PIPE IN “PREVIOUS TREATMENT” FROM S5d]**

**[PN: SCALE]**

1. Not at all effective
2. Extremely effective

[PN: SINGLE CODE]

D13. How long did your doctor tell you to take your **[IF** OTC NAÏVE, SHOW **“current medication”, IF Rx USER, SHOW “current prescription medication”, IF Rx LAPSED, SHOW “previous medication”]?**

(Please enter the number in weeks/days)

Your current medication is: **[PN: PIPE IN “CURRENT TREATMENT” VARIABLE CREATED AFTER S5c]**

**[IF Rx LAPSED SHOW**: Your previous medication was: **[PIPE IN “PREVIOUS TREATMENT” FROM S5d]**

- ______ weeks [NUMERIC. RANGE 0–53]

OR

- ______ days [NUMERIC. RANGE 0–31]
- I purchased my current medication myself / without a doctor’s recommendation **[PN:** IF OTC NAÏVE]
- I don’t remember [PN: EXCLUSIVE]

D14. Thinking about treatments for trouble sleeping, which of the following **aspects** are the **most important** to you? Please first select the **most important one** to you and then other important ones.

**[PN: TOP]**

- **a. The most important aspect [PN: SINGLE CODE]**
- **b. Other important aspects [PN: MULTICODE – UP TO 4]**

**[PN: LEFT STATEMENTS]**

1. To fall asleep quickly
2. To sleep a reasonable number of hours
3. To not wake up throughout the night
4. To wake up easily in the morning
5. To wake up feeling refreshed and restored
6. To help my brain switch off at night
7. To not feel the strain on my mood, concentration or patience
8. To wake up feeling ready for a new day
9. To feel like my real self
10. To not have nightmares at night
11. To repair my normal sleeping pattern / habits
12. To not cause dependency
13. To not make me feel groggy the next day
14. Other, please specify ____________ **[PN: FIX]**
15. None of the above **[PN: FIX. EXCLUSIVE]**

[PN: RANDOMIZE]

**D15.** **[IF NON-Rx LAPSED, SHOW]** To what extent does your **[IF** OTC NAÏVE, SHOW **“current medication”, Rx USER, SHOW “current prescription medication”]** meet your expectations?

**[IF Rx LAPSED, SHOW]** To what extent did your **previous prescription medication** meet your expectations?

(Please select one answer per statement)

[Your current medication is: **[PN: PIPE IN “CURRENT TREATMENT” VARIABLE CREATED AFTER S5c]**

**[IF Rx LAPSED SHOW**: Your previous medication was: **[PIPE IN “PREVIOUS TREATMENT” FROM S5d]**

**[PN: TOP RESPONSES]**

1. Not meeting my expectations at all

2.

3.

4.

5.

6.

7. Fully meeting my expectations

**[PN: LEFT, PIPE IN THE EXPECTATIONS FROM D14a AND D14b]**

**[PN: SINGLE CODE PER ROW]**

D17. We would like to understand the **impact of insomnia on your daily** life when you take your current medication vs. when you do not take your current medication.

Please use the list below to indicate the level of impact for:

[PN: TOP, RESPONSES]

- a. Nights when you **have taken** medication
- b. Nights when you **have NOT taken** medication

**[PN: LEFT, SCALE]**

1. No impact at all
2. Extremely high impact

**[PN: SINGLE CODE PER COLUMN]**

**SECTION E: Patient–Doctor relationship**

E1. Which type of doctor are you currently seeing regarding your trouble sleeping?

(Please selected only one answer)

*If you are seeing more than one doctor, please select the main doctor who helps you managing your trouble sleeping.*

1. Family doctor / Primary care physician / General practitioner
2. Neurologist
3. Psychiatrist
4. Sleep specialist
5. Psychologist
6. Internal medicine physician
7. Other (please specify ____________) **[PN: ANCHOR]**

[PN: RANDOMIZE. SINGLE CODE]

E2. Thinking about your **[PIPE IN CODE SELECTED AT E1]**, how would you rate their level of knowledge about insomnia and its treatments?

**[PN: SCALE]**

1. Not at all knowledgeable about insomnia and its treatment
2. Extremely knowledgeable about insomnia and its treatment

**E3.** In general, how confident are you with recommendations made by your **[PIPE IN CODE SELECTED AT E1]?**

(Please place yourself on the scale below)

**[PN: SCALE]**

1. Not at all confident
2. Extremely confident

**E4.** To what extent do you agree or disagree with each of the following statements?

(Please select one answer only per each statement)

[PN: TOP, RESPONSES]

- Strongly disagree
- Neither agree nor disagree
- Strongly agree

[PN: LEFT, STATEMENTS]

1. I sometimes feel that my doctor is not really listening to my concerns
2. I have to be very proactive to get the best care from my doctor
3. I often hesitate to honestly discuss my trouble sleeping with my doctor
4. I believe my doctor is an expert in managing trouble sleeping
5. My doctor treats me as a partner in management of my trouble sleeping
6. It is important to me to have a good relationship with my doctor
7. I don’t feel my doctor takes my insomnia seriously
8. My doctor takes the time to get to the bottom of my insomnia
9. I feel my doctor takes the full impact of my insomnia into consideration when making decisions
10. My doctor prescribes a medication too quickly without fully exploring other options
11. I feel my doctor is very focused on improving my quality of life

**[PN: SINGLE CODE PER STATEMENT. RANDOMIZE STATEMENTS]**

E5. Please read the pair of statements below and indicate where you would place yourself

between statements*.*

I am the main person making decisions about my treatment for insomnia | | My doctor is the main person making decisions about my treatment for insomnia

[PN: SLIDING SCALE, PLEASE START AT NEUTRAL]

# HCP quantitative survey questionnaire

Note: The survey also included a series of questions related to an investigational product, described as “Product X”, which are not shown here.

**Section A: Insomnia caseload**

Firstly, we would like to understand a bit more about your caseload of insomnia patients.

A2. You said that [PN: INSERT ANSWER FROM S8] of your patients suffer from insomnia.

On a weekly basis, what percentage of your time is spent treating insomnia?

(Please type in your answer)

- Range 0–100%

[OPEN NUMERIC. 0–100%]

A3. Of the [INSERT ANSWER AT S9_1] adult patients that you treat for insomnia with prescription drugs in a typical month, what proportion have no underlying condition causing their insomnia?

(Please type in your answers)

[PN: OPEN NUMERIC RANGE 0 TO 100 FOR EACH ROW]

1. Patients with no underlying condition causing insomnia that has been identified
2. Patients with a diagnosed underlying condition causing insomnia
3. Patients with a suspected underlying condition causing insomnia

[PN: OPEN NUMERIC RANGE 0–100% PER ANSWER OPTION. ANSWERS MUST SUM TO 100%]

A4. What proportion of the insomnia patients you treat fall under the following categories?

(Please type in your answer next to each insomnia type)

[PN: OPEN NUMERIC RANGE 0 TO 100 FOR EACH ROW]

1. Chronic insomnia (has lasted longer than 3 months)
2. Acute insomnia (has lasted less than 3 months)
3. Unknown duration

[PN: OPEN NUMERIC RANGE 0–100% PER ANSWER OPTION. ANSWERS MUST SUM TO 100%]

A5. Thinking about all the insomnia patients you currently personally manage, what proportion are also being treated for a mental health condition?

(Please type in your answer)

- Range 0–100%

[OPEN NUMERIC. 0–100%]

**Section B: Perception of insomnia**

B1. To what extent do you agree or disagree with each of the following statements about **insomnia**?

(Please use the sliders to select one answer per statement)

[PN: USE CUSTOM SET-UP, STATEMENTS SHOWN ONE AT A TIME WITH SLIDER TO ANSWER]

[PN: TOP, RESPONSES]

1. Strongly disagree
2. Strongly agree

[PN: LEFT, STATEMENTS]

1. I have a personal interest in insomnia and/or the science of sleep
2. I always formally diagnose insomnia in my patients
3. It is critical to treat insomnia proactively irrespective of the comorbidities / underlying conditions present
4. Insomnia is often a symptom linked to another underlying condition
5. Insomnia needs to be addressed as quickly as possible in most of my patients
6. Given the other conditions of my patients, insomnia is less urgent to treat
7. I always ask my patients about their current sleep pattern
8. I often refer my insomnia patients to other physicians
9. In most cases I see, insomnia is typically a lifestyle issue rather than a medical one
10. Insomnia is one of the most complex conditions I treat
11. Insomnia is a significantly undertreated condition
12. I believe insomnia is primarily a transient condition rather than a chronic disorder in its own right
13. I tend to distinguish between acute and chronic insomnia patients
14. Most of my patients are proactive in reporting their insomnia
15. Insomnia is one of the most rewarding conditions I treat
16. I am viewed as a thought leader by other physicians for the treatment of insomnia
17. I regularly consult with other physicians around the difficult-to-treat insomnia cases I have

[PN: SINGLE CODE PER STATEMENT, RANDOMISE STATEMENTS.]

**B2.** Please read the pair of statements below and tell us where you would place your view on insomnia diagnosis between them.

| I make an insomnia diagnosis mostly based on my impression of the patient | ˂---------------------------------˃ | I make an insomnia diagnosis mostly based on what the patient tells me |
| --- | --- | --- |

[PN: SLIDING SCALE, PLEASE START AT NEUTRAL]

B3. Compared to most other conditions you treat, how challenging do you find insomnia to be?

[PN: SCALE]

1. Not challenging at all
2. Extremely challenging

[PN: SINGLE CODE]

B4. Compared to most other conditions you treat, how confident are you in managing insomnia effectively?

[PN: SCALE]

1. I do not feel confident at all
2. I feel very confident

[PN: SINGLE CODE]

B5. Thinking about most of the insomnia patients you see, please rate the level of impact that insomnia has on their **ability to carry out their normal daily activities.**

[PN: SCALE]

1. No impact on patient’s ability to carry out normal daily activities
2. Significant impact on patient’s ability to carry out normal daily activities

[PN: SINGLE CODE]

B6. Thinking about most of the insomnia patients you see, please rate the level of impact that insomnia has on their **mental wellbeing.**

[PN: SCALE]

1. No impact on patient’s mental wellbeing
2. Significant impact on patient’s mental wellbeing

[PN: SINGLE CODE]

**Section C: Attitudes towards insomnia management**

C1. Once you have established that a patient has insomnia, for what proportion of patients do you take the following as your action(s) in the first consultation?

(Please type in your answer next to each potential actions)

[PN: NUMERIC]

1. I check if they have already made lifestyle changes (e.g. limiting caffeine, limiting screen time) and if not, I ask them to do this first
2. I offer them Cognitive Behavioural Therapy for Insomnia (CBT-I)
3. I suggest an over-the-counter (OTC) treatment
4. I offer them a prescription medication
5. Suggest referral to a psychologist
6. Suggest referral to another specialist (e.g. a psychiatrist, neurologist or sleep specialist)

[PN: ONE % PER ROW – RANGE 0–100% PER ROW – CAN BE EQUAL TO OR MORE THAN 100%]

C2. In cases in which you don’t recommend a prescription medication as the first step, how long do you suggest patients try with lifestyle changes and/or OTC medication before you will consider prescription medications?

[PN: NUMERIC RANGE 0–50] weeks for lifestyle changes

[PN: NUMERIC RANGE 0–50] weeks for OTC medication

[PN: ADD OPTION EXCLUSIVE: I don’t typically send a patient away without a prescription medication] IF SELECTED DON’T SHOW NEXT QUESTION

C3. In cases in which you don’t recommend a prescription medication and suggest patients try with lifestyle changes and/or OTC medication as the first step, do you typically set up a follow-up appointment with these patients?

1. Yes – I will suggest a follow-up appointment
2. No – I wait for the patient to come back to me

[PN: SINGLE CODE]

C4. Please rate your agreement with the following statements related to insomnia management using the scale below.

[PN: USE CUSTOM SET-UP, STATEMENTS SHOWN ONE AT A TIME WITH SLIDER TO ANSWER]

[PN: TOP, RESPONSES]

1. Strongly disagree
2. Strongly agree

[PN: LEFT, RESPONSES]

1. CBT-I is an effective treatment for most patients who go through it
2. I only prescribe insomnia treatment when my patient asks for it
3. Insomnia doesn’t require prescription treatment as it is not a life-threatening condition and it generally improves on its own
4. I try to avoid prescription medication for insomnia as it often introduces new problems for my patients such as side effects and dependency

[PN: SINGLE CODE PER STATEMENT, RANDOMISE STATEMENTS]

C5. Thinking about the patients you manage for insomnia, please indicate your agreement with the following statements.

[PN: USE CUSTOM SET-UP, STATEMENTS SHOWN ONE AT A TIME WITH SLIDER TO ANSWER]

[PN: TOP, RESPONSES]

1. Strongly disagree
2. Strongly agree

[PN: LEFT, RESPONSES]

1. I believe the majority of my insomnia patients are committed to following my advice regarding lifestyle changes
2. I adapt my treatment decision based on whether I can trust the patient to follow my advice
3. To effectively manage insomnia, I need my patients to take more responsibility in making lifestyle changes and improving their sleep hygiene
4. Most of my insomnia patients are in desperate need for an immediate relief before they can consider long-term management options
5. Most of my insomnia patients are reluctant to seek professional help until the quality of life burden becomes too great for them to manage
6. I believe some of my patients will be on insomnia prescription drugs for the majority of their life
7. For patients I initiate on prescription therapy for insomnia, I always start with the same prescription drug
8. I always manage insomnia more proactively for patients who have a hectic lifestyle
9. Rapid improvement in patient’s sleep is one of the most important factors when treating insomnia

[PN: SINGLE CODE PER STATEMENT, RANDOMISE STATEMENTS]

**Section D: Attitudes toward insomnia treatments**

In the next section, we would like to better understand your approach with insomnia treatments.

D1. Approximately what proportion of your insomnia patients fall into each of the following categories in terms of their current treatment?

(Note: one patient might be in more than one category)

1. Insomnia patients applying lifestyle changes/improvements to sleep hygiene
2. Insomnia patients receiving a Cognitive Behavioural Therapy for insomnia, such as CBT-I
3. Insomnia patients using self-guided sleep or relaxation online programs/apps
4. Insomnia patients receiving an OTC treatment
5. Insomnia patients receiving a prescription medication
6. Insomnia patients receiving other types of intervention/treatment

[PN: OPEN NUMERIC ANSWER PER ROW, RANGE 1–100%, SUM CAN BE GREATER THAN 100]

D2. To what extent do you think each of these treatment options can make a positive impact on your patients’ ability to sleep?

[PN: TOP, SCALE]

1. Very low impact
2. Very high impact

[PN: LEFT, STATEMENTS]

1. Lifestyle changes/improvements to sleep hygiene
2. Cognitive Behavioural Therapy for insomnia, such as CBT-I
3. Self-guided sleep or relaxation online programs/apps
4. OTC treatments
5. Prescription medications

[PN: SINGLE CODE PER STATEMENT, SHOW STATEMENTS IN SAME ORDER AS PREVIOUS TABLE]

D3. To what extent do you agree or disagree with each of the following statements regarding your overall approach to insomnia treatment?

(Please select one answer per statement)

[PN: SCALE, USE CUSTOM SET-UP, STATEMENTS SHOWN ONE AT A TIME WITH SLIDER TO ANSWER]

[PN: TOP, RESPONSES]

1. Strongly disagree
2. Strongly agree

[PN: LEFT, STATEMENTS]

1. When managing insomnia, I always try to delay the initiation of prescription medication as much as possible
2. My approach to insomnia treatment is rarely influenced by patients’ psychiatric comorbidities (like depression, anxiety)
3. Lifestyle changes and good sleep hygiene alone can resolve the majority of insomnia cases
4. Overall improvement in sleep quality is more important than speed of sleep onset
5. I always refer to treatment guidelines when selecting insomnia treatments
6. I believe some benefits from insomnia treatment can be attributed to a placebo effect
7. I would prefer an insomnia treatment with better efficacy even if it comes with some additional risk of side effects

[PN: SINGLE CODE PER STATEMENT, RANDOMISE STATEMENTS]

D4. For each pair of statements below, please indicate where your overall treatment approach for your insomnia patients lies on the scale.

**[PN: SCALE]**

|  | I have a preferred prescription treatment approach that I use for most of my insomnia patients | ˂---------------------------------˃ | I tend to tailor my prescription treatment approach for individual insomnia patients |
| --- | --- | --- | --- |
|  | My patients are usually the ones to initiate discussions about prescription medication | ˂----------------------------------˃ | I am usually the one to initiate discussions about prescription medication |
|  | Most of my insomnia patients can be effectively managed without prescription medication | ˂---------------------------------˃ | Most of my insomnia patients can only be effectively managed with prescription medication |
|  | I evaluate the benefit of an insomnia treatment by how well the patient sleeps during the night | ˂---------------------------------˃ | I evaluate the benefit of an insomnia treatment by how well the patient functions the next day |
|  | Most of my patients need prescription drugs for their insomnia treatment | ˂---------------------------------˃ | Over-the-counter (OTC) drugs alone are usually effective in treating insomnia |
|  | Treating the underlying condition causing insomnia often resolves insomnia | ˂---------------------------------˃ | Insomnia often needs to be treated as an independent medical condition |
|  | In most cases, the key reason to treat insomnia is to improve patients’ quality of life | ˂---------------------------------˃ | In most cases, the key reason to treat insomnia is to alleviate its impact on other comorbidities |

[PN: SLIDING SCALE, PLEASE START AT NEUTRAL, ONE PAIR AT A TIME. RANDOMISE THE PAIRS]

D5. To what extent do you agree or disagree with each of the following statements on the relationship with your insomnia patients and the insomnia treatments you use?

[PN: SCALE, USE CUSTOM SET-UP, STATEMENTS SHOWN ONE AT A TIME WITH SLIDER TO ANSWER]

[PN: TOP, RESPONSES]

1. Strongly disagree
2. Strongly agree

[PN: LEFT, STATEMENTS]

1. If I believe that an insomnia treatment is right for my patient, I will work hard to convince him/her to try it even if they are initially reluctant
2. I am willing to prescribe an insomnia treatment option that my patient requests, even when that treatment would not be my first choice
3. If a patient is not satisfied with an insomnia treatment, I switch treatment immediately
4. I am reluctant to discuss insomnia treatments associated with a risk of dependency with my patients
5. I am willing to stop treatment immediately if I feel a patient is becoming too reliant on their medication
6. I get frustrated when patients insist that I prescribe a medication treatment for their insomnia
7. It is important for my patients to be confident that they will fall asleep quickly after taking their medication
8. It is important for my patients to be confident that they will stay asleep during the whole night after taking their medication
9. I feel my patients expect insomnia treatments to provide an immediate solution to their insomnia
10. I leave it up to my patients to take their sleep medications as and when they need to
11. I generally tell my patients only to take their sleep medication when absolutely necessary
12. I believe most of my patients follow my advice regarding prescription medication
13. If a drug does not provide immediate relief, many of my patients will stop taking it

[PN: SINGLE CODE PER STATEMENT, RANDOMISE STATEMENTS]

NEW SCREEN

D7. Please rank your most important treatment goals while managing insomnia patients with prescription medication (a) for acute insomnia patients (lasting less than 3 months) and (b) for chronic insomnia questions (lasting longer than 3 months)

[PN: RANKING DRAG AND DROP WITH 2 COLUMNS AND THE HEADINGS ‘ACUTE INSOMNIA PATIENTS’ AND ‘CHRONIC INSOMNIA PATIENTS’ ON EACH COLUMN, USE QUESTION LIBRARY FI030]

1. Improve sleep quality over time
2. Improve sleep duration
3. Improve daytime functioning over time
4. Successfully manage associated side effects
5. Increase patient satisfaction with their sleep
6. Increase patient satisfaction with their overall quality of life
7. Get the patient off prescription medication as quickly as possible
8. Increase in number of nights with “natural sleep” (sleep without prescription medication)
9. Control insomnia symptoms while the underlying mental health condition is treated
10. Provide patient with relief while they are making lifestyle changes
11. Find a balance between lowest possible dose and frequency and patient ability to function
12. Other (please specify) [PN: ANCHOR]

[PN: DRAG AND DROP ALL STATEMENTS – SHOW RANKING # IN DROP BOX – ALLOW RESPONDENTS TO CHANGE ORDER IN DROP BOX]

D8. For each pair of statements below, please indicate where your overall view lies on the scale.

|  | I am confident in managing medication dependency among my insomnia patients | ˂---------------------------------˃ | I find it difficult to manage medication dependency among my insomnia patients |
| --- | --- | --- | --- |

[PN: SLIDING SCALE, PLEASE START AT NEUTRAL, ONE PAIR AT A TIME. RANDOMISE THE PAIRS]

D9. To the best of your knowledge what proportion of your insomnia patients on prescription medication fall into the following categories?

1. Patients who are dependent (both physically and/or psychologically) on their prescription medication **[Range 0–100%]**
2. Patients who are at risk of becoming dependent (both physically and/or psychologically) on their prescription medication **[Range 0–100%]**
3. Patient not at risk of becoming dependent **[Range 0–100%]**

[PN: NUMERIC, RANGE 0 TO 100% TOTAL MUST EQUAL TO 100%]

E10. To what extent do you agree or disagree with each of the following statements regarding discontinuing prescription medication for your insomnia patients?

[PN: SCALE, USE CUSTOM SET-UP, STATEMENTS SHOWN ONE AT A TIME WITH SLIDER TO ANSWER]

[PN: TOP, RESPONSES]

1. Strongly disagree
2. Strongly agree

[PN: LEFT, STATEMENTS]

1. Once a patient has started sleeping well, I try to slowly get them off insomnia prescription drugs
2. Once an underlying mental health condition causing insomnia is resolved, I immediately reduce the dosing frequency of insomnia prescription medication
3. As long as the patient is not requesting repeat prescriptions too frequently, I am comfortable prescribing sleep medication over a longer period of time
4. I tell my patients to take their medication only for the period necessary but leave it to them to tell me when they feel ready to come off it
5. I always advise my patients to not take insomnia prescription drugs for longer periods
6. Even for chronic insomnia patients, I prefer not to prescribe insomnia prescription drugs for a longer period
7. Most of my patients prefer not to use prescription drugs for insomnia treatment

# Results from patient quantitative survey

**Table A1. Patient quantitative survey: Living with insomnia – knowledge**

| **Question** | **Response options** | **Proportion**  **(n=700)** |
| --- | --- | --- |
| Which of the following expressions would you use to define what “good sleep” means to you? | Having undisturbed sleep  Waking up feeling refreshed  Able to fall asleep naturally  Falling asleep within a short period of time from going to bed  Waking up and feeling energetic all the next day  Waking up feeling ready for the day  Waking up and not feeling drowsy all the next day  Having more than a certain number of hours of sleep  Waking up and looking rested and rejuvenated  Waking up at a reasonable time, not very early in the morning  Sleeping without nightmares | 63%  59%  52%  46%  45%  39%  38%  34%  34%  25%  20% |
| Which of the following do you consider to be the main reason for your current insomnia? Please consider any potential triggers for your current insomnia. | Racing thoughts at night  Recent stressful life events or circumstances  Hard to unwind at night  There is no apparent cause  Stressful experiences in my past  Other mental health issues  Hectic lifestyle  Poor sleep routine  Too much screen time on digital devices  It’s just who I am  A recent traumatic event, e.g. bereavement  Fear of nightmares  I don’t know | 39%  36%  35%  31%  28%  17%  16%  16%  15%  13%  11%  6%  3% |
| Thinking about when you first started having sleeping problems (before talking to a healthcare professional), what caused you to start thinking of your trouble sleeping as a problem? | Experiencing symptoms more often/with greater intensity than usual  Not being able to cope with the effect of insomnia in my everyday life  Desire to have better options to resolve my trouble sleeping  Encouragement from my family to take better care of myself and manage my trouble sleeping  Seeing or hearing a commercial related to trouble sleeping  Seeing posts on social media about trouble sleeping  None of the above | 62%  55%  45%  28%  12%  7%  3% |
| When you were looking for information about insomnia, which of the following sources did you use or visit? | **HCPs**  Discussion with my PCP/GP  Discussion with a Specialist  Discussion with pharmacists  **Online sources**  Specialist sleep websites  Online forums and blog posts  Social media  Online patient networks or support groups  **Other influential people**  Discussion with family and friends  Discussion with alternative health practitioner  Discussion with staff at health shops  **Advertising and media**  Magazine / Newspaper articles  Books  Brochures found in my doctor’s office | **90%**  75%  44%  29%  **56%**  34%  22%  15%  13%  **56%**  41%  17%  14%  **36%**  16%  16%  14% |

Values shown reflect the percentage of patients selecting each answer.

**Table A2. Patient quantitative survey: Living with insomnia – impact**

| **Question** | **Response options** | **Proportion**  **(n=700)** |
| --- | --- | --- |
| Impact of and worries about insomnia – to what extent do you agree with these statements? | People who do not experience insomnia cannot understand what it means  I am often frustrated with my trouble sleeping  I am hopeful that my trouble sleeping will get better  Trouble sleeping is my biggest health concern  I feel supported by my family and friends when I need to manage my insomnia  I will probably always need prescription medications to manage my trouble sleeping  I feel guilty about how my trouble sleeping affects people around me  I worry my insomnia is seen by others as a mental health issue  I am reluctant/self-conscious to admit to others that I have insomnia  I feel my insomnia is under control | 59%  57%  56%  34%  33%  31%  26%  24%  17%  11% |

Values shown reflect the percentage of patients selecting each answer.

**Figure A1. Patient quantitative survey: Impact of insomnia on life and personality (n=700)**


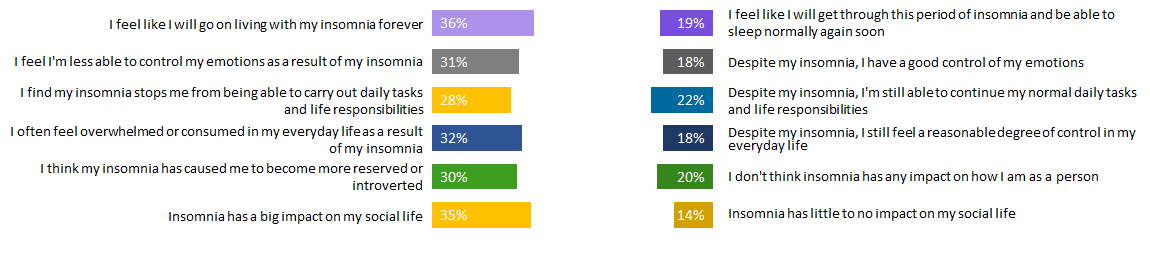


Values reflect the percentage of respondents selecting the lowest 2 scores (1 or 2) and the highest 2 scores (6 or 7) on a 1- to 7-point scale, where the bottom end of the scale (1) means strong agreement with the left-hand item and the top end of the scale (7) means strong agreement with the right-hand item.

**Table A3. Patient quantitative survey: Living with insomnia – self-management**

| **Question** | **Response options** | **Proportion**  **(n=700)** |
| --- | --- | --- |
| Insomnia vs other medical issues* | I believe my trouble sleeping is an independent medical problem, not influenced by any other health issues | 31% |
| Which of the following types of measures, if any, have you tried over the past 3 months to improve your sleep? | Talking to a doctor or psychologist to help manage your trouble sleeping  Taking a medication treatment that you need a prescription for  Taking over-the-counter medication that you can buy from the pharmacy without a prescription  Implementing lifestyle changes (healthy diet, avoiding caffeine, exercise, etc.)  Talking to an HCP to understand the underlying condition  Establishing a sleep routine (e.g. avoiding daytime sleep, setting regular bed & wake times)  Taking herbal tea/homemade remedies  Monitoring sleep with a device or an app (Sleep, Fitbit, Pillow, Sleep Genius, Apple Watch, etc.)  Using Cognitive Behaviour Therapies for insomnia (CBT-I) | 77%  67%  58%  57%  56%  53%  48%  26%  7% |
| Which, if any, of the following have you ever used to manage your insomnia by yourself? (Please select all that apply) | Reducing caffeine intake  Reading before bed  Not looking at phones or screens before bed  Running or other physical activities  Warm bath before bed  Alcohol  Cannabis (in any form)  Weighted blanket  Acupuncture  Diet  Relaxation  Music  Other | 76%  69%  57%  48%  45%  28%  23%  15%  11%  1%  1%  1%  1% |
| Which, if any, of the following mindfulness methods have you ever used to manage your insomnia by yourself? (Please select all that apply) | Breathing exercises  Meditation  Yoga  Cognitive Behavioural Therapy for Insomnia  Sleep maintenance/Structured sleep rituals  Other | 66%  50%  34%  23%  1%  2% |
| Which, if any, of the following ways do you currently use to monitor your sleep quantity and quality? | Smart Devices, such as FitBit, Smart Watches and Oura Ring  Sleep Tracking Apps downloaded onto my phone  A Diary (paper or digital)  Other  None of the above | 33%  29%  17%  1%  46% |
| Which, if any, of the following ways have you previously used to monitor your sleep quantity and quality? | Smart Devices, such as FitBit, Smart Watches and Oura Ring  Sleep Tracking Apps downloaded onto my phone  A Diary (paper or digital)  Other  None of the above | 17%  22%  35%  1%  44% |

Values shown reflect the percentage of respondents selecting each answer except those marked *, which reflect the percentage of respondents selecting the highest 2 scores (6 or 7) on a 1- to 7-point scale, where 7 indicates the strongest agreement.

**Figure A2. Patient quantitative survey: Management and control of insomnia (n=700)**


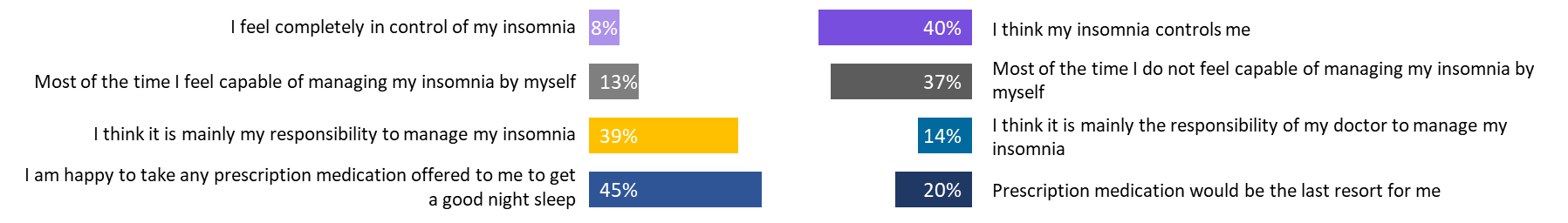


Values reflect the percentage of respondents selecting the lowest 2 scores (1 or 2) and the highest 2 scores (6 or 7) on a 1- to 7-point scale, where the bottom end of the scale (1) means strong agreement with the left-hand item and the top end of the scale (7) means strong agreement with the right-hand item.

**Table A4. Patient quantitative survey: Patient–doctor relationship**

| **Question** | **Response options** | **Proportion**  **(n=700)** |
| --- | --- | --- |
| Which of the following motivated the decision to see a doctor? | I had been suffering for a while and I needed help  My insomnia got worse, and I needed help  Not being able to cope with the effect of insomnia in my everyday life  I had tried a lot of measures myself with little success  I wanted to see what the doctor would say  I wanted to know if something was wrong with me  I did not see the doctor for my insomnia, I booked a consultation for another reason and then we ended up discussing my sleep  Encouragement from my family to take better care of myself and manage my trouble sleeping  A friend or family member suggested I speak to my doctor  My research of information sources made me think reaching out to a doctor would help | 48%  47%  35%  32%  31%  28%  18%  18%  17%  13% |
| Which type of doctor are you currently seeing regarding your trouble sleeping? | Family doctor/PCP/GP  Psychiatrist  Sleep specialist  Psychologist  Neurologist  Internal medicine physician  Homeopath  Other | 61%  12%  11%  7%  6%  1%  0%  1% |
| After how many times did you see your doctor about your trouble sleeping before your first prescription medication specifically for insomnia? | 1  2  3  4  5  6–10  11+  I don’t know | 27%  19%  15%  3%  4%  3%  1%  28% |
| Opinion on HCP’s level of care and knowledge | It is important to me to have a good relationship with my doctor  I feel my doctor is very focused on improving my quality of life  I feel my doctor takes the full impact of my insomnia into consideration when making decisions  My doctor takes the time to get to the bottom of my insomnia  My doctor treats me as a partner in management of my trouble sleeping  I believe my doctor is an expert in managing trouble sleeping  I have to be very proactive to get the best care from my doctor  My doctor prescribes a medication too quickly without fully explore other options  I sometimes feel that my doctor is not really listening to my concerns  I often hesitate to honestly discuss my trouble sleeping with my doctor  I don’t feel my doctor takes my insomnia seriously | 71%  52%  48%  47%  37%  35%  28%  17%  16%  16%  15% |

**Figure A3. Patient quantitative survey: Patient–doctor relationship (n=700)**


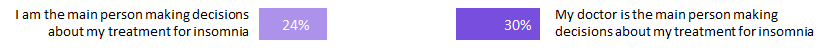


Values reflect the percentage of respondents selecting the lowest 2 scores (1 or 2) and the highest 2 scores (6 or 7) on a 1- to 7‑point scale, where the bottom end of the scale (1) means strong agreement with the left-hand item and the top end of the scale (7) means strong agreement with the right-hand item.

**Table A5. Patient quantitative survey: Treatments and medications**

| **Question** | **Response options** | **Proportion (n=700)** |
| --- | --- | --- |
| Thinking about your current/previous prescription medication, and your insomnia, to what extent do you agree with the following statements?* | I would really like to/have liked to take insomnia medications less often  I have/had asked my doctor about taking my insomnia medication less often  I have/had a love-hate relationship with my insomnia medication  Even with my current insomnia medication I cannot/could not sleep properly  I feel/felt that my insomnia can be effectively resolved in other ways  I feel my current insomnia medication is/was not the right one for me | 50%  32%  28%  23%  19%  16% |
| Most important aspects for treatment | To wake up feeling refreshed and restored  To sleep a reasonable number of hours  To fall asleep quickly  To repair my normal sleeping pattern / habits  To not wake up throughout the night  To help my brain switch off at night | 15%  13%  11%  11%  10%  10% |
| Average length of time taking current treatment | <1 month  1–6 months  7–12 months  13–24 months  25–36 months  37–48 months  49+ months | 9%  34%  14%  11%  6%  4%  9% |
| Thinking about your current/previous medication to what extent do you agree with the following statements? | I always take/took medications following advice from my doctor / as prescribed  I cannot/could not imagine going on holiday/‌going away without my medication  My medication is/was the only thing that helps me to sleep  I am keen/I was keen to stop my medication soon, but I am worried that I could not sleep  I use/used my medication as little as possible, only when it is really necessary  If I have/had good sleep for a couple of nights, I will skip my medication  I am not/I was not comfortable talking about my medication with others around me  I avoid/avoided using my medication as much as possible | 62%  45%  41%  34%  30%  28%  21%  16% |

Values shown reflect the percentage of patients selecting each answer.

# Results from HCP quantitative survey

**Table A6. HCP quantitative survey: Perceptions of insomnia**

| **Question** | **Response options** | **Proportion**  **(n=723)** |
| --- | --- | --- |
| Attitude towards insomnia | In most cases I see, insomnia is typically a lifestyle issue rather than a medical one  I regularly consult with other physicians around the difficult-to-treat insomnia cases I have  Insomnia is one of the most rewarding conditions I treat  I am viewed as a thought leader by other physicians for the treatment of insomnia  Given the other conditions of my patients, insomnia is less urgent to treat  I believe insomnia is primarily a transient condition rather than a chronic disorder in its own right  I often refer my insomnia patients to other physicians | 16%  14%  12%  9%  6%  6%  5% |
| Attitude towards insomnia diagnosis | I make an insomnia diagnosis mostly based on what the patient tells me | 44% |
| Perception of the challenge of insomnia | I find insomnia to be extremely challenging to treat | 24% |
| Perceived impact of insomnia on daily activities | Significant impact on patient’s ability to carry out normal daily activities | 49% |
| Perceived impact of insomnia on mental wellbeing | Significant impact on patient’s mental wellbeing | 70% |

Values shown reflect the percentage of respondents selecting the highest 2 scores (6 or 7) on a 1- to 7-point scale, where 7 indicates the strongest agreement.

**Table A7. HCP quantitative survey: Attitudes towards insomnia management**

| **Question** | **Response options** | **Proportion**  **(n=723)** |
| --- | --- | --- |
| Confidence in managing insomnia* | I feel very confident about managing insomnia | 22% |
| Perceptions of insomnia patients and insomnia patient management* | To effectively manage insomnia, I need my patients to take more responsibility in making lifestyle changes and improving their sleep hygiene  Rapid improvement in patients’ sleep is one of the most important factors when treating insomnia  I adapt my treatment decision based on whether I can trust the patient to follow my advice  Most of my insomnia patients are in desperate need for an immediate relief before they can consider long-term management options  Most of my insomnia patients are reluctant to seek professional help until the quality-of-life burden becomes too great for them to manage | 50%  32%  31%  26%  25% |
| Once you have established that a patient has insomnia, for what proportion of patients do you take the following as your actions in the first consultation? | I check if they have already made lifestyle changes (e.g. limiting caffeine, limiting screen time) and if not, I ask them to do this first  I offer them a prescription medication  I suggest an over-the-counter treatment  I offer them Cognitive Behavioural Therapy for Insomnia  Suggest referral to a psychologist  Suggest referral to another specialist (e.g. a psychiatrist, neurologist or sleep specialist) | 73%  37%  26%  23%  17%  13% |
| In cases in which you don’t recommend a prescription medication as the first step, how long do you suggest patients try with lifestyle changes and/or OTC medication before you will consider prescription medications? | Time suggested for lifestyle changes (mean)  Time suggested for OTC medication (mean) | 5.2 weeks  4.3 weeks |
| In cases in which you don’t recommend a prescription medication and suggest patients try with lifestyle changes and/or OTC medication as the first step, do you typically set up a follow-up appointment with these patients? | Yes – I will suggest a follow-up appointment  No – I wait for the patient to come back to me | 86%  14% |
| Confidence in managing medication dependency* | I am confident in managing medication dependency among my insomnia patients | 17% |

Values shown reflect the percentage of respondents selecting each answer except those marked *, which reflect the percentage of respondents selecting the highest 2 scores (6 or 7) on a 1- to 7-point scale, where 7 indicates the strongest agreement.

**Figure A4. HCP quantitative survey: To what extent do you think each of these treatment options can make a positive impact on your patients’ ability to sleep? (n=723)**


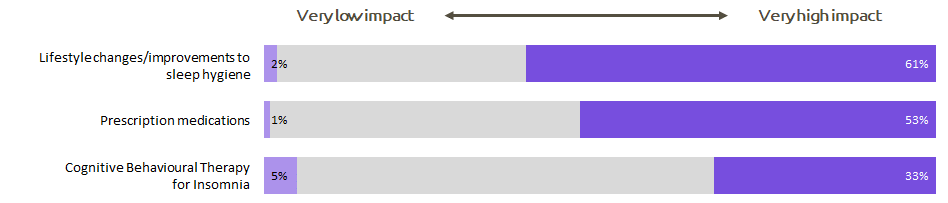


Values reflect the percentage of respondents selecting the lowest 2 scores (1 or 2) and the highest 2 scores (6 or 7) on a 1- to 7‑point scale, where the bottom end of the scale (1) means strong agreement with the left-hand item and the top end of the scale (7) means strong agreement with the right-hand item.

**Figure A5. HCP quantitative survey: Attitudes towards treatment (n=723)**

**
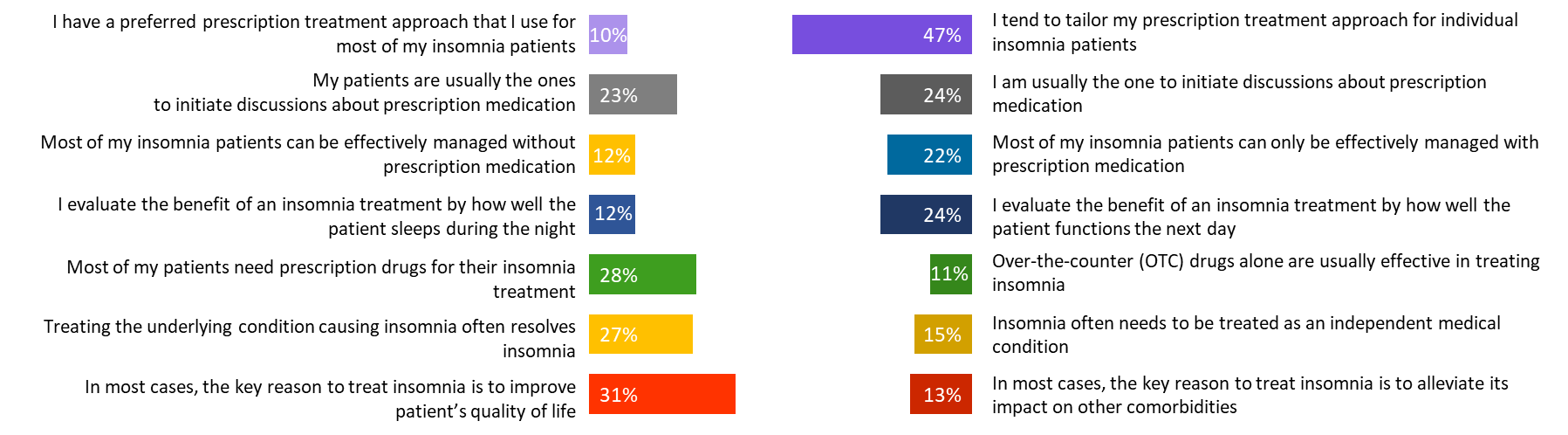
**

Values shown reflect the percentage of respondents selecting the lowest 2 scores (1 or 2) and the highest 2 scores (6 or 7) on a 1- to 7-point scale, where the bottom end of the scale (1) means strong agreement with the left-hand item and the top end of the scale (7) means strong agreement with the right-hand item.

**Table A8. HCP quantitative survey: Attitudes towards insomnia treatments**

| **Question** | **Response options** | **Proportion**  **(n=723)** |
| --- | --- | --- |
| Attitudes towards relationship with insomnia patients and treatment* | I get frustrated when patients insist that I prescribe a medication treatment for their insomnia  I believe most of my patients follow my advice regarding prescription medication  I am reluctant to discuss insomnia treatments associated with a risk of dependency with my patients  I leave it up to my patients to take their sleep medications as and when they need to  If a patient is not satisfied with an insomnia treatment, I switch treatment immediately  I am willing to prescribe an insomnia treatment option that my patient requests, even when that treatment would not be my first choice | 21%  19%  18%  12%  10%  8% |
| Most important attributes when assessing insomnia prescription medication for chronic insomnia | Does not cause dependency  Safety for long-term use  Ability to improve daytime functioning  No negative impact on cognition  Ability to improve sleep over time | 54%  44%  42%  38%  34% |
| Thinking about insomnia prescription medication, what are the main challenges (outside your control) you are facing at the moment? (Please do not consider treatment safety, efficacy or treatment-related factors in this question) | Lack of long-term options for some patients (due to guidelines restrictions)  Patient reluctance  Lack of clarity in guidelines  Limitation in my ability to prescribe certain insomnia medications  Pharmacists/hospital pharmacy challenging my treatment decision  Side effects/tolerability concerns  Dependency  Other  None of the above | 64%  44%  32%  27%  14%  1%  1%  1%  10% |
| Attitude towards discontinuing prescription medication* | Once a patient has started sleeping well, I try to slowly get them off insomnia Rx drugs  I always advise my patients to not take insomnia Rx drugs for longer periods  Once an underlying mental health condition causing insomnia is resolved, I immediately reduce the dosing frequency of insomnia Rx medication  Even for chronic insomnia patients, I prefer not to prescribe insomnia Rx drugs for a longer period  I tell my patients to take their medication only for the period necessary but leave it to them to tell me when they feel ready to come off it  As long as the patient is not requesting repeat Rxs too frequently, I am comfortable prescribing sleep medication over a longer period of time  Most of my patients prefer not to use Rx drugs for insomnia treatment | 43%  37%  24%  19%  16%  15%  8% |
| There are significant unmet needs in the current prescription medication options for these types of insomnia patients | Patients with chronic insomnia (lasting longer than 3 months)  Elderly patients  Active patients (working professional and/or demanding lifestyle)  Non-active patients (retired, non-working and/or relaxed lifestyle)  Patients with mental health conditions (e.g. depression, anxiety)  Patients with perceived higher risk of dependency  Patients with acute insomnia (lasting less than 3 months | 66%  62%  60%  57%  55%  55%  47% |
| Unmet needs in insomnia treatment | Does not cause dependency  Safe for long-term use  Does not cause morning grogginess  Ability to improve sleep over time  Ability to improve daytime functioning | 66%  63%  43%  42%  38% |

Values shown reflect the percentage of respondents selecting each answer except those marked *, which reflect the percentage of respondents selecting the highest 2 scores (6 or 7) on a 1- to 7-point scale, where 7 indicates the strongest agreement.

Rx, prescription medication.

**Figure A6. HCP quantitative survey: Efficacy/safety priority when choosing insomnia prescription medication by patient group (n=723)**


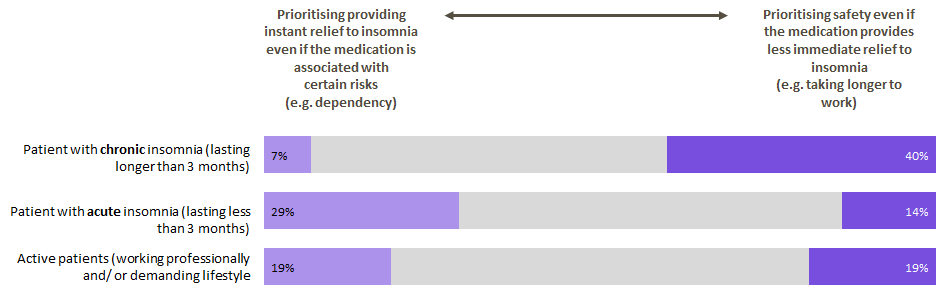
 Values shown

Values reflect the percentage of respondents selecting the lowest 2 scores (1 or 2) and the highest 2 scores (6 or 7) on a 1- to 7-point scale, where the bottom end of the scale (1) means strong agreement with the left-hand item and the top end of the scale (7) means strong agreement with the right-hand item.
